# Selectivity by Small-Molecule Inhibitors of Protein Interactions Can Be Driven by Protein Surface Fluctuations

**DOI:** 10.1371/journal.pcbi.1004081

**Published:** 2015-02-23

**Authors:** David K. Johnson, John Karanicolas

**Affiliations:** 1 Center for Computational Biology, University of Kansas, Lawrence, Kansas, United States of America; 2 Department of Molecular Biosciences, University of Kansas, Lawrence, Kansas, United States of America; Heidelberg Institute for Theoretical Studies (HITS gGmbH), GERMANY

## Abstract

Small-molecules that inhibit interactions between specific pairs of proteins have long represented a promising avenue for therapeutic intervention in a variety of settings. Structural studies have shown that in many cases, the inhibitor-bound protein adopts a conformation that is distinct from its unbound and its protein-bound conformations. This plasticity of the protein surface presents a major challenge in predicting which members of a protein family will be inhibited by a given ligand. Here, we use biased simulations of Bcl-2-family proteins to generate ensembles of low-energy conformations that contain surface pockets suitable for small molecule binding. We find that the resulting conformational ensembles include surface pockets that mimic those observed in inhibitor-bound crystal structures. Next, we find that the ensembles generated using different members of this protein family are overlapping but distinct, and that the activity of a given compound against a particular family member (ligand selectivity) can be predicted from whether the corresponding ensemble samples a complementary surface pocket. Finally, we find that each ensemble includes certain surface pockets that are not shared by any other family member: while no inhibitors have yet been identified to take advantage of these pockets, we expect that chemical scaffolds complementing these “distinct” pockets will prove highly selective for their targets. The opportunity to achieve target selectivity within a protein family by exploiting differences in surface fluctuations represents a new paradigm that may facilitate design of family-selective small-molecule inhibitors of protein-protein interactions.

## Author Summary

Despite intense interest and considerable effort, there are few examples of small molecules that directly inhibit protein-protein interactions. Crystal structures of early successes have highlighted the plasticity of the protein surface, as some inhibitor-bound proteins are captured in conformations that are distinct from both their unbound and their protein-bound conformations. The lack of a single well-defined structure presents a challenge for predicting the members of a protein family to which a given compound will show activity (ligand selectivity). Here we generate ensembles of conformations from simulation, and show that we can predict ligand selectivity based on which family members sample conformations complementary to the ligand. This approach may present a new avenue for designing highly-selective inhibitors of protein-protein interactions.

## Introduction

Selectivity of a compound for its desired protein target—or targets—is an important property optimized in the course of small-molecule drug discovery [1]. Some diseases, such as chronic myeloid leukemia, can be traced to dysfunction of a single protein target (BCR-ABL); in such cases, drugs such as imatinib are sought to act selectively against that target [2]. Conversely, a number of drugs (such as sunitinib and chlorpromazine) have proven exceedingly successful because they act on multiple targets [3,4]; this has led to increased interest in “polypharmacology” to address complex disease states such as cancer and psychiatric conditions [5]. A clear downside of compound promiscuity, however, is the potential for adverse effects (toxicity) due to interactions with unrelated proteins, or even interactions with other proteins in the same family as the target [6]. One recent cautionary example is ABT-263 (navitoclax), a Bcl-2 inhibitor that exhibited a dose-limiting adverse effect (thrombocytopenia) stemming from its inhibition of Bcl-xL [7,8].

Tuning the selectivity of a lead compound can sometimes be carried out by exploiting differences in shape and electrostatics between target and off-target proteins, using insights from structure-activity relationships (SAR) [9] or structural biology [10–12] to focus on features that will prevent specific undesirable interactions. While determinants of selectivity have been carefully mapped in a number of “traditional” drug targets, such as kinases, this has not yet been the case for emerging classes of “non-traditional” drug targets, most notably small-molecule inhibitors of protein-protein interactions.

Though extensive efforts to identify inhibitors of protein interactions have only recently begun to bear fruit, structural analysis of available examples has revealed that often the inhibitor-bound protein is captured in a conformation that is distinct from both the unbound and protein-bound conformations [13]. In such cases, the unbound and protein-bound conformations could not have served to rationalize binding of the inhibitor its desired target, let alone explain selectivity against other members of the protein family.

We recently described an approach for biased exploration of protein fluctuations, in order to better sample pocket-containing conformations at protein interaction sites [14]. We found that when starting from an unbound protein structure, we observe low-energy conformations that include deep surface pockets at druggable sites but not elsewhere on the protein surface. These findings supported a “conformational selection” model [15], whereby inhibitors recognize low-lying excited states of the protein that are naturally visited under physiological conditions.

A natural implication of this conformational selection model is that the particular variety of pocket shapes visited by the protein surface will dictate the regions of chemical space in which complementary inhibitors can be found: this would have clear implications for designing new inhibitors. Conversely, one may instead view this complementarity from the perspective of the *ligand*: under this model, an inhibitor is expected to be active against a given protein if, and only if, the protein surface includes a suitably complementary pocket among those that are sampled under physiological conditions. Within a protein family, then, only the subset of family members that sample the corresponding pocket will be inhibited by this compound.

The Bcl-2 protein family in humans is comprised of about 25 members that together regulate apoptosis through a series of protein-protein interactions that induce either pro-death (Bax, Bak, and others), pro-survival (Bcl-xL, Bcl-2, Mcl-1, Bcl-w, and others), or derepression/sensitizing (Bid, Bim, and others) activity [16]. The critical step of disregulating apoptosis in tumor maintenance and chemoresistance has made certain members of this family exceptionally attractive targets for therapeutic intervention for many years, and in a variety of cancers [17]. Despite the overall dearth of examples of small molecules that inhibit protein interactions, a number of such compounds have been reported against assorted members of the Bcl-2 family. Further, selectivity of these inhibitors against a panel of Bcl-2 family members have also been reported in some cases. These data, together with the availability of multiple experimentally-derived structures of inhibitor-bound complexes, position the Bcl-2 family as a rich model system in which we can explore the determinants of selectivity for small-molecule inhibitors of protein-protein interactions.

Here, we use the Bcl-2 family to explore whether the ensemble of pocket shapes sampled on protein surfaces can be used to explain ligand selectivity. We will use simulation to generate ensembles of pocket-containing conformations [14], then directly probe whether a complementary pocket for a given ligand is present in the ensemble. If successful, this approach may allow us ultimately to predict, and by extension *design*, inhibitors with a desired selectivity profile; such compounds could serve both as a starting point for development of new therapeutics, and also as “tool compounds” to probe the underlying biological mechanism of disease.

## Methods

Computational methods are implemented in the Rosetta software suite [18]; Rosetta is freely available for academic use (www.rosettacommons.org), with the new features described here included in the 3.6 release. Computational methods are summarized below, with further detail (including Rosetta command-lines to access these tools) is available in the *Supporting Methods* section.

### Identifying surface pockets

Pockets were identified using the “pocket” protocol in Rosetta [14]. This is a local pocket detection approach adapted from the Ligsite algorithm [19], and it uses geometric criteria to identify concave regions on a protein surface suitable for small-molecule binding. The approach entails building a small grid in the vicinity of one or two “target” residues, then mapping the van der Waals surface for the whole protein onto the grid; the exposed grid points are marked as “solvent.” Next, the grid is screened to identify linear segments of solvent points that are bounded by protein points; the grid points along these segments are marked as “pocket” points. Pocket grid points are clustered into “pockets,” and then any clustered pockets not in direct contact with the target residue(s) are discarded. The “deep volume” of a pocket is defined as the volume of the pocket that is well-sequestered from the solvent (more than 2.5 Å from any solvent grid point).

**Fig 1:**
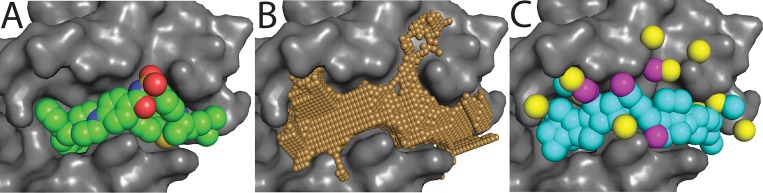
Building “exemplars” from surface pockets. (A) Bcl-xL (*grey surface*) is shown in complex with an inhibitor (*spheres*). (B) The protein surface features a large pocket (*small white spheres*) that is complementary in shape to the inhibitor. (C) From this surface pocket, an “exemplar” is built: a map of the “perfect” ligand to complement this protein surface, without considerations of atom connectivity. The exemplar is comprised of hydrogen bond donors (*yellow*) and acceptors (*magenta*) that complement surface polar groups on the protein, and hydrophobic atoms that fill the remainder of the surface pocket (*cyan*). In the studies we report here, we will use the shape and chemical features of the exemplar as a proxy for the shape and chemical features of the protein surface pocket.

Generation of a representative protein surface pocket is demonstrated in **Fig. 1**. A number of related approaches have been described for identifying potential small molecule binding sites on protein surfaces in this way [19–25]; the primary difference between these methods relative to the method described here is our use of “deep” pocket volume, which leads to improved discrimination when distinguishing known ligand binding sites from other shallow concave regions on the protein surface [14]. These “deep” pocket volumes are correlated to, but smaller than, pocket volumes identified by other representative methods [14]; in the context of this study, then, we expect that focusing our pocket description on regions in direct contact with the protein will allow the most critical features to be captured with minimal extraneous information.

### Target residue selection

The selection of the target residue pairs is important in determining the location of the grid, and in turn may affect the resulting pocket shapes. To avoid biasing results towards any particular inhibitor—and to allow the method to be applied in cases for which no inhibitor has yet been identified—we did not use the structure of the protein in complex with any small-molecule ligand when defining the target residues.

Instead, we developed an algorithm to select suitable target residues starting from the structure of the protein-protein complex. First, the “Robetta” server [26,27] is used to estimate the contribution to the binding free-energy of each interfacial sidechain (ΔΔGres) via computational alanine scanning. All pairs of interfacial residues are then exhaustively tested by building a 24 Å candidate grid placed at the residue pair’s center of mass, and summing ΔΔGres for each residue that falls on this grid. To ensure that the grid placement captures the key energetic contributors to the protein-protein interaction, the residue pair that captures the largest cumulative ΔΔGres is used in subsequent studies. In essence, this approach aims to align the pocket grid such that it is optimally close to the energetically dominant residues of the protein-protein interaction. This tool is implemented in the Rosetta software suite, and its use it demonstrated in the *Supporting Methods* section [18].

The effect of the particular target residues used will be examined further in the *Results* section.

### Building ensembles of pocket-containing conformations

Ensembles of pocket containing conformations were generated using the “relax” protocol in Rosetta, which incorporates both backbone and side chain degrees of freedom in a Monte Carlo search. Starting from the standard Rosetta energy function, we added a term corresponding to the “deep pocket” volume multiplied by a proportionality constant. This biasing potential favors pocket-containing conformations that have more deep pocket volume, and thus drives sampling towards such conformations.

In our previous work, we found that applying a proportionality constant of -0.25 Rosetta energy units per Å^3^ allowed identification of pocket-containing conformations with similar energies to those observed in unbiased simulations [14]. In the present study, therefore, we reused the same proportionality constant. A separate independent trajectory was used to generate each of the 1000 output conformations used for each protein included in our studies. It took about 5 minutes to generate each conformation on a modern CPU, though the independence of trajectories made it trivially scalable to multiple processors.

### Generating “exemplars” to represent pockets

To compare the shape and chemical similarity of surface pockets, we introduce the concept of an exemplar: a map of the “perfect” ligand that could complement a surface pocket, without the natural constraints of bond connectivity or chemistry. The lack of such physical requirements means that the exemplar does not correspond to any particular chemical entity, but instead simply consists of a collection of atoms in space. This collection occupies volume, and may include hydrogen bond donors and acceptors, but by construction does not imply any particular connectivity between the atoms.

Exemplars are built from the “deep volume” that defines a protein surface pocket. First, the ideal location for a hydrogen bond partner of every donor/acceptor on the protein surface is determined; if this location lies within the pocket, then this region of the pocket volume is reserved for the polar group that will complement the protein surface. After placing these polar groups in the pocket, the remaining unoccupied volume is filled with hydrophobic (carbon) atoms using a greedy algorithm, such that the center of two atoms are no less than 1.7 Å apart. Exemplar points are then clustered together based on a proximity threshold of 5 Å, so that cases in which two small pockets flank the target residues are represented by a single exemplar. Generation of an exemplar from a representative protein surface pocket is demonstrated in **Fig. 1**; complete details of our implementation are included in S1 Text.

These exemplars are analogous in philosophy to the popular pharmacophore maps used in medicinal chemistry to reflect consensus properties of known ligands [28,29]; while the latter approaches rely on mimicry of the existing binding partners [30–33], however, exemplars are instead built purely from features of the protein surface. Recently, the utility of protein-based pharmacophores has been explored for pose prediction and virtual screening [34–36], but such approaches have not been used for the comparison of pocket shapes as described below.

### Comparing exemplars

Having represented the shape and chemical features of a protein surface pocket by its “perfectly complementary ligand” (the exemplar), we are now positioned to quantitatively compare pockets using standard tools developed for comparison of chemical entities in 3D, provided that these tools do not require (or assume) knowledge of bond connectivity. A variety of ligand-based shape comparison methods have been developed for aligning pairs of molecules on the basis of volume overlap [37,38]; a convenient modern implementation of this approach is the ROCS software (OpenEye Scientific Software, Santa Fe, NM) [39,40]. ROCS represents molecular shape as the sum of Gaussians centered at each atomic position, and can rapidly calculate the near-optimal alignment that maximizes volume overlap between two molecules. Chemical groups are also included as separate Gaussians in the scoring/optimization step, to include electrostatic effects.

The results we present below are built upon quantitative comparisons of the similarity in shape and hydrogen bonding patterns for pairs of protein surface pockets. In all cases we carry out this analysis by generating an exemplar for each of the two pockets, then using ROCS to align and score the overlap between these exemplars.

We note that method to define pockets, and thus exemplars, makes use of a grid centered at a pair of target residues. To quantitatively examine the effect of the grid orientation on the exemplar similarity, we used four pocket-containing crystal structures and rotated each to 100 random orientations. Using one of these structures as a reference (ABT-737 bound to Bcl-xL), we found that rotated variants of this structure produced similar exemplars, as did rotated variants of a related structure (Bcl-xL in complex with a related ligand). In contrast, rotated variants of Bcl-xL bound to unrelated ligands, or rotated variants of Mcl-1, produced highly dissimilar exemplars (**S1 Fig.**). Collectively, these observations demonstrate that the orientation dependence of generating exemplars on a grid makes a negligible contribution to the exemplar distances we report below.

## Results

The principal goal of the studies we report here is to understand how conformational changes at a protein interaction site can drive selectivity of the small-molecule inhibitors that bind at these sites. We have developed an approach for building an “exemplar”—a map of an idealized ligand—that describes a protein surface pocket and enables quantitative comparisons between pockets (**Fig. 1**, *Methods* section). In each of the sections below, we use exemplar comparisons to examine the similarity of pockets observed on the surface of proteins comprising the Bcl-2 family.

### Plasticity of the protein surface allows recognition of diverse chemical scaffolds

There have been dozens of reported inhibitors of Bcl-2 family members [7,8,41–59], spanning a broad range of chemotypes. Unfortunately, experimentally-derived structures are not available for the majority of these inhibitors in complex with their cognate protein partner(s); this makes it difficult to gain insight into the detailed basis for molecular recognition in these cases. Instead, we began by compiling a comprehensive collection of all structures in the Protein Data Bank containing a Bcl-2 family member in complex with a non-fragment small-molecule inhibitor bound at the protein interaction site: these are listed in **S1 Table**. There are 28 such complexes, covering 26 unique inhibitors, and 3 different proteins are represented: Bcl-xL (14 structures), Bcl-2 (9 structures), and Mcl-1 (5 structures).

**Fig 2:**
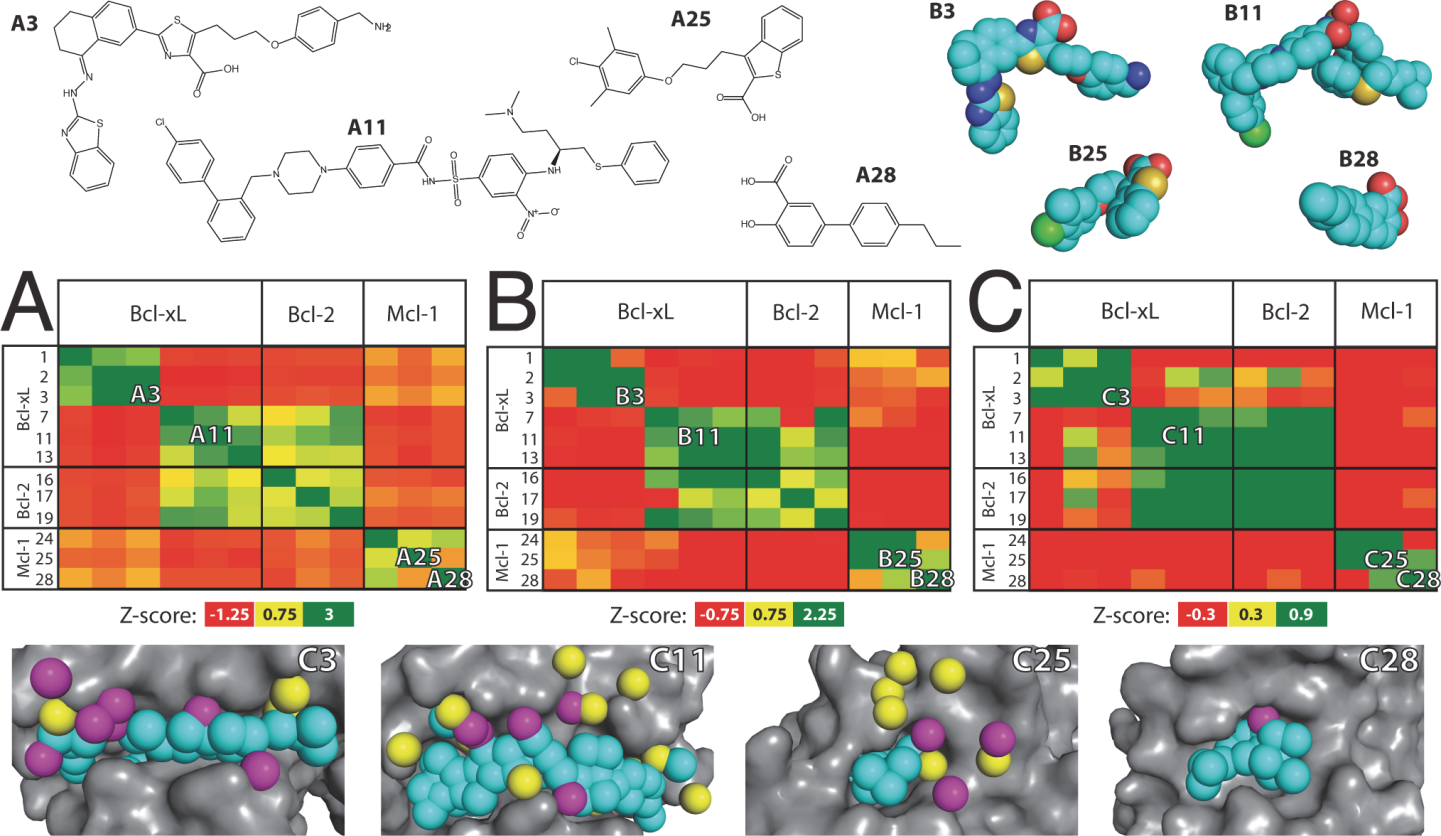
Bcl-2 family members recognize different inhibitors using distinct surface pockets. In all cases color gradient indicates the similarity between complexes, expressed as Z-scores (*green* are most similar, *red* are most dissimilar). (A) Chemical similarity of the inhibitors. (B) Three-dimensional similarity of the inhibitors’ active conformation. (C) Similarity of protein surface pockets, measured using exemplar similarity. Numbering in all cases corresponds to complexes in **S1 Table**. A representative subset of the complexes are included in this figure; a corresponding figure containing all available complexes is available as **S2 Fig**.

The diversity of the inhibitors in this set is readily apparent from the Tanimoto similarity of fingerprints describing each chemical structure (**Fig. 2A**). Unsurprisingly, groups of compounds that are similar by this measure typically represent a chemical series designed by a single research group (e.g. compounds ***1–3*** from WEHI [60]). Given this collection of chemical scaffolds, we sought to ask how such diverse compounds could be recognized on the surface of a single protein family. There are two possibilities: either these compounds might adopt a shared three-dimensional structure not evident from their chemical structure (i.e. a non-obvious example of “scaffold hopping” [61]), or else the protein surface must be sufficiently malleable to adopt different conformations when binding different ligands.

To test whether these distinct compounds present a common structure to complement the protein surface, we carried out comparisons of the inhibitors’ shape and chemical features in their active (bound) conformations (**Fig. 2B**). To examine the pattern of similarity between chemical structure and three-dimensional structure we sorted the all-vs-all Tanimoto scores for each set, and found a statistically significant non-zero Spearman correlation coefficient between these rankings (p < 10^-35^). This observation confirms that dissimilar chemical structures do *not* somehow adopt a shared three-dimensional structure. As expected, a given shape and pattern of hydrogen bond donors/acceptors is conserved within a chemical series, but not across different chemical scaffolds.

To directly evaluate similarity of the conformations adopted by the protein to bind each ligand, we next carried out an analogous comparison using the exemplar derived from each inhibitor-bound pocket (**Fig. 2C**). Here again we observe the same pattern of similarity, mimicking both that of the chemical structures (p < 10^-26^) and their corresponding three-dimensional structures (p < 10^-20^). While each of the inhibitors within a given chemical class bind to a very similar pocket on the surface of the cognate protein, different chemical classes each take advantage of a dramatically different pocket on the protein surface. Moreover, the surfaces of different proteins bound to similar ligands (e.g. Bcl-xL complex ***7*** vs Bcl-2 complex ***19***) resemble one another more closely than the surface of a single protein bound to chemically distinct ligands (e.g. Bcl-xL complexes ***3*** vs ***7***).

These observations highlight the plasticity of this protein surface: multiple members of the Bcl-2 family can adopt similar conformations to bind a given ligand, yet a given protein can also form radically different surface pockets to accommodate different ligands. Together with the fact that the unbound structures of these Bcl-2 family members lack suitably deep surface pockets for inhibitor binding (**S3 Fig.**), the observations presented here underscore the fact that molecular recognition in this protein family cannot be explained using a single conformation, but rather an explanation of ligand selectivity will instead require consideration of the many available conformations that this surface can adopt.

### Ensembles of low-energy pocket-containing conformations

To explore pocket-containing conformations of these proteins, we generated conformational ensembles using simulations in the presence of a biasing potential [14]. In essence, energy associated with the biasing potential in these simulations serves as a proxy for the binding energy of some (unspecified) ligand, which in turn serves to stabilize alternate conformations of the protein surface. The biasing potential takes account of the protein surface geometry (“deep pocket volume”), but does not encode any information about the identity or features of any particular ligand. Thus, this approach allows efficient sampling of the conformational space available for protein reorganization in response to ligand binding. While other methods could have been employed to sample alternative conformations (such as unbiased molecular dynamics simulations with retrospective analysis to identify pocket-containing conformations [62]), we chose this approach because it allowed us to rapidly generate a large ensemble of pocket containing conformations.

As noted earlier, our approach defines the relevant protein surface on the basis of the protein/peptide interaction site, and not based on any known small-molecule inhibitor (*Methods* section). In light of the fact that particular “target” residues are needed to produce the ensembles of pocket-containing conformations, we begin by examining the effect of the target residues on the resulting “pocket ensemble.”

**Fig 3:**
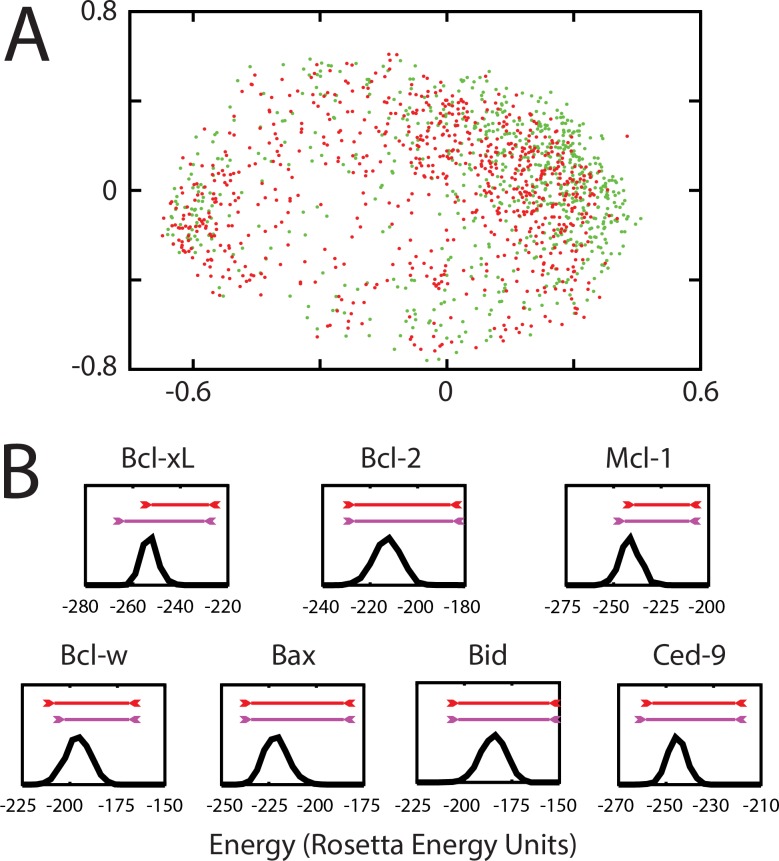
Ensembles of low-energy pocket-containing conformations. (A) To examine the effect of the particular target residues used in generating the ensemble of pocket-containing conformations, we generated separate ensembles from the top-scoring pair of target residues (Ala93 and Arg139, *green*) and the second-best pair (Ala93 and Ala142, *red*). We use exemplars to compare the similarity of surface pockets on each conformation, and we show each conformation on the two-dimensional projection that best reflects the pairwise distances between them. The overlap between the two ensembles highlights the robustness of the conformations to the particular target residues. (B) For each member of the Bcl-2 family, we generated an ensemble of conformations from unbiased simulations; the distribution of these energies is shown (*black*). The range of energies for pocket-containing conformations generated using a biasing potential target residues derived from the Bcl-xL protein interaction site (*magenta*) or the Mcl-1 protein interaction site (*red*) suggest that many of these conformations are energetically accessible to these proteins under physiological conditions. All energies shown here were evaluated in the absence of the biasing potential, for fair comparison. Each simulation is started from the structure of the unbound protein; a corresponding figure starting from the peptide-bound structures containing all available complexes is available as **S5 Fig**.

Starting with the Bcl-2 family member Bcl-xL, we selected the two top-scoring pairs of target residues resulting from analysis of its peptide-bound structure. We then used each pair of target residues to generate separate ensembles of 2000 pocket-containing conformations, with each trajectory initiated from the unbound structure of Bcl-xL. To visually compare the conformational “pocket space” sampled by these two ensembles we built an exemplar from each pocket, and used multidimensional scaling analysis (MDS) to construct the two-dimensional projection that best reflects the pairwise distance between every pair of exemplars. The resulting map demonstrates that these two ensembles are strongly overlapping, and points to the robustness of the conformational space sampled to the particular target residues used (**Fig. 3A**). In light of the similarity between the ensembles, we used conformations generated using the second-best scoring pair of target residues (rather than the top-scoring pair), for consistency with our previous studies of Bcl-xL [14].

While application of the same approach for selecting target residues led to similar residues for most members of the Bcl-2 family, the residue pair selected on Mcl-1 was notably different. While this derived exclusively from different energetic contributions to the protein-protein interaction from each protein surface, it is interesting to note that known inhibitors of Bcl-xL and Mcl-1 bind at slightly different regions on the surface of these two proteins (**S4 Fig.**). To ensure that the range of pockets across the Bcl-2 family would be fully captured in our studies, we used *both* the pair of residues derived from Bcl-xL and those derived from Mcl-1 to generate ensembles of pocket-containing conformations; in the analyses presented below, all such conformations are combined into a single ensemble regardless of the target residues used to generate them.

To ensure that only physiologically relevant conformations were included in the subsequent analysis, we compared their energies to those obtained in equivalent unbiased simulations of the corresponding protein. Previously, we found that this method produced ensembles of pocket-containing conformations a slightly higher but overlapping distribution of energies than the unbiased ensemble [14]. With the caveats that conformations generated in our “pocket opening” protocol are not drawn from a Boltzmann distribution and the energy differences do not necessarily capture real differences in free energy, we instead simply collect pocket-containing conformations from the biased simulations that are within 15 Rosetta energy units of the unbiased ensemble. Thus, these represent conformations that are in principle available within the unbiased ensemble, but are simply not observed due to limitations of sampling. The distribution of energies observed in an unbiased simulation of each Bcl-2 family member is presented in **Fig. 3B**, along with the range of energies spanned by conformations from biased simulations.

### Ensembles of conformations sampled by Bcl-2 family members include the inhibitor-bound pockets

**Fig 4:**
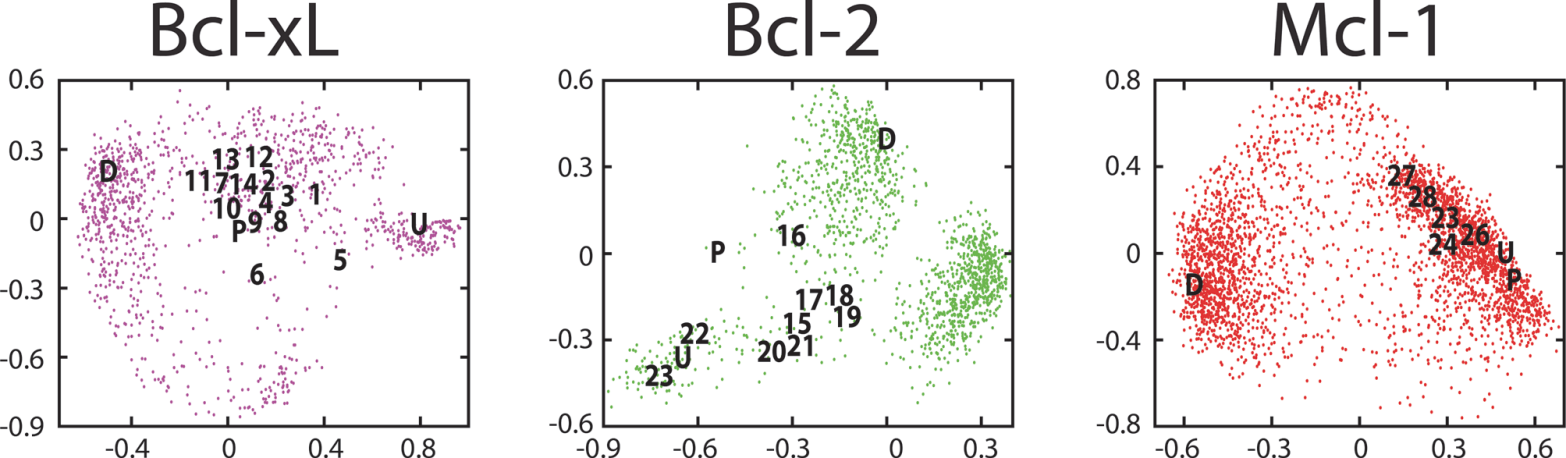
Maps of “pocket space” sampled by individual Bcl-2 family members. The ensemble of pockets observed from simulation: individual conformations are represented as points on a two-dimensional projection that reflects the pairwise distances between their exemplars. The relative position of exemplars from experimentally-derived Bcl-xL unbound (“*U*”) and peptide-bound (“*P*”) structures are indicated, as are the positions of exemplars from Bcl-xL structures solved in complex with various inhibitors (numbers correspond to complexes listed in **S1 Table**). Exemplars marked “*D*” correspond to the same “distinct” conformations described in **Fig. 5**.

We next sought to compare the surface pockets on conformations comprising these ensembles to those pockets observed on experimentally-derived inhibitor-bound structures. We again turned to MDS analysis, this time including exemplars built from the experimentally-derived structures of the unbound, peptide-bound, and inhibitor-bound protein. For each of Bcl-xL, Bcl-2, and Mcl-1 (all family members for which structures of inhibitor-bound complexes are available), the resulting maps show that the pocket-containing conformational space sampled via simulation includes thorough coverage of experimentally-derived inhibitor-bound structures (**Fig. 4**). Though each simulation was initiated from either the unbound or the peptide-bound crystal structure, the resulting ensemble is notably distinct from these starting states; instead, the biasing potential drove sampling towards pocket-containing conformations that include examples very similar to the inhibitor-bound structures. In addition, each protein also samples a collection of conformations (marked “*D*”) with exemplars that differ from those observed in any available inhibitor-bound conformations: these will be discussed in more detail in the following section.

**Fig 5:**
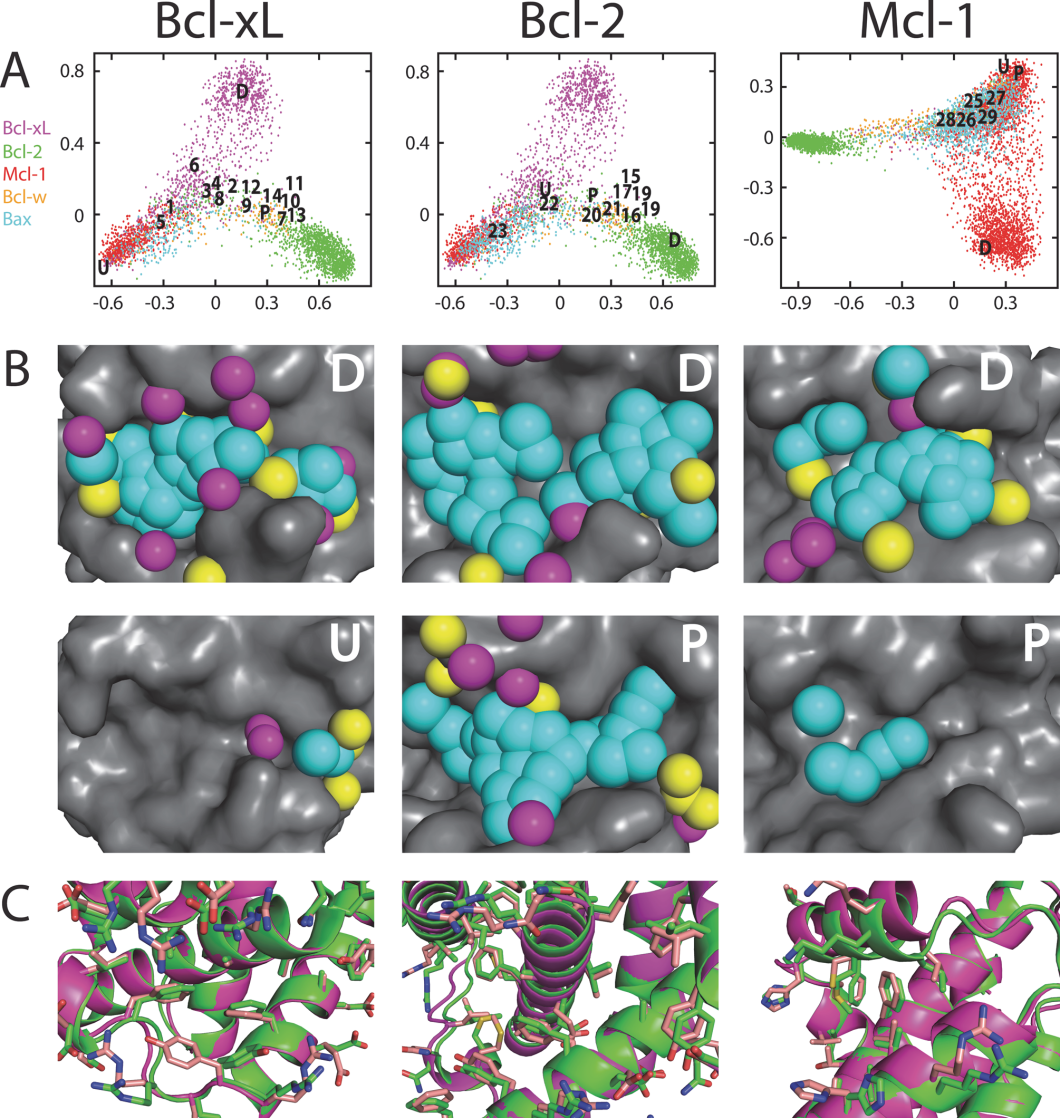
Comparison of “pocket space” sampled by each Bcl-2 family member. (A) A projection built using ensembles collected from simulations of several Bcl-2 family members: Bcl-xL (*magenta*), Bcl-2 (*green*), Mcl-1 (*red*), Bcl-w (*orange*), and Bax (*cyan*). Bid and Ced-9 were used in generating the projection, but for clarity are not included on this map. Target residues derived from the Bcl-xL protein interaction site were used in generating exemplars shown on the Bcl-xL and Bcl-2 MDS plots, whereas target residues derived from the Mcl-1 protein interaction site were used in generating exemplars shown on the Mcl-1 MDS plots. For each of Bcl-xL, Bcl-2, and Mcl-1 we observe a distinct region of conformational space (“*D*”) that is not sampled by any other Bcl-2 family member. (B) Comparison of an exemplar from each “distinct” region to the corresponding unbound (“*U*”) or peptide-bound (“*P*”) protein structure from which the simulation was initiated. (C) Comparison of the conformation harboring the “distinct” pocket to the corresponding unbound/peptide-bound protein structure from which the simulation was initiated.

In all three cases we find that these ensembles, generated without any prior information about the inhibitors, span the space of known inhibitors. This indicates that each protein is predisposed to adopt the particular pocket shapes observed in the corresponding inhibitor-bound structures: these are not conformational changes that are “induced” by the inhibitor, but rather these are among a suite of available conformations from which the inhibitor may select. Upon binding, meanwhile, smaller changes to the protein surface may then occur in response to particular features of the ligand (such as reorientation of hydrogen bond donors and acceptors).

### Different Bcl-2 family members sample different ensembles of surface pockets

To explore differences between ensembles of pocket-containing conformations of Bcl-2 family members, we carried out an analogous MDS analysis comparing exemplars from multiple family members; an important aspect of exemplar generation and comparison is that the description of a surface pockets is not tied to the sequence of the protein, allowing one to compare exemplars on the surfaces of different proteins. We compiled conformations generated from simulations of each Bcl-2 family member (Bcl-xL, Bcl-2, Mcl-1, Bcl-w, Bax, Bid, and Ced-9), and carried out MDS analysis using the complete set of exemplars (**Fig. 5A**). Unsurprisingly, we observe that different family members sample different surface pockets; however, we do not observe such differences between the (closely related) human and mouse Mcl-1 sequences (**S6 Fig.**).

In these projections we again note that the ensemble generated for a given protein spans the majority of known inhibitors of that protein. Unsurprisingly, given the similarity we observed between inhibitor-bound Bcl-2 pockets and inhibitor-bound Bcl-xL pockets (**Fig. 2C**), we find that most pockets observed in the inhibitor-bound structures occupy a similar region on this projection (all except ***1***, ***5***, ***6***, and ***23***). Notably, many known inhibitors fall in a region of “pocket space” that is sampled by more than one protein. In the case of Bcl-xL, for example, many known inhibitors bind to pockets that are not only observed in simulations of Bcl-xL, but also in simulations of Bcl-2. On this projection, these inhibitors (all except ***1***, ***5***, and ***6***) reside in a region sampled by both Bcl-xL and Bcl-2. Similarly, most of the Bcl-2 inhibitors (all except ***23***) are in the same region sampled by both Bcl-xL and Bcl-2; in contrast, this other Bcl-2 inhibitor (***23***) overlaps with a regions sampled by Mcl-1 but not Bcl-xL.

In addition to regions shared by more than one family member, each map contains a “distinct” region that is sampled exclusively by a single protein (**Fig. 5A**, “*D*”). As expected, exemplars corresponding to these conformations are very different from those of the (unbound or peptide-bound) conformations from which the corresponding simulations were initiated (**Fig. 5B**). Further comparison of the conformations themselves show that these alternate conformations are accessed primarily through concerted reorganization of the sidechains that comprise the surface pocket, though corresponding changes to the protein backbone—especially in the case of Mcl-1—are also required to enable this reorganization (**Fig. 5C**). In summary, relatively modest structural changes to the protein conformation can produce radically different exemplars, and none of the inhibitors described to date bind to these particular protein conformations. To facilitate further study of these alternate protein conformations, we have made several such representatives publicly available on Proteopedia [63,64] (http://proteopedia.org/wiki/index.php/User:John_Karanicolas/Selectivity_by_small-molecule_inhibitors_of_protein_interactions_can_be_driven_by_protein_surface_fluctuations, with the first model for each protein corresponding to the conformation we highlight here).

The regions that we have described as “distinct” on these maps correspond to pockets that are only sampled by a single member of the Bcl-2 family. In other words, these “distinct” pockets are far from each of the pockets sampled by other family members. So far, we have identified these regions visually on the basis of a two-dimensional projection of “pocket space”; to avoid the loss of information associated with reduction of dimensionality, however, we can instead identify “distinct” pockets using exemplar distances directly.

For each conformation comprising the ensembles used above, we found the exemplar distance of the closest pocket sampled by a different family member. To avoid describing rarely-sampled (outlying) conformations as distinct, we subtracted from this the exemplar distance of the closest pocket from one’s own ensemble. This measure, that we will call “distinctness,” is largest for conformations taken from regions of pocket space that are well-sampled within a given ensemble, but not visited in the ensembles of the other family members.

**Fig 6:**
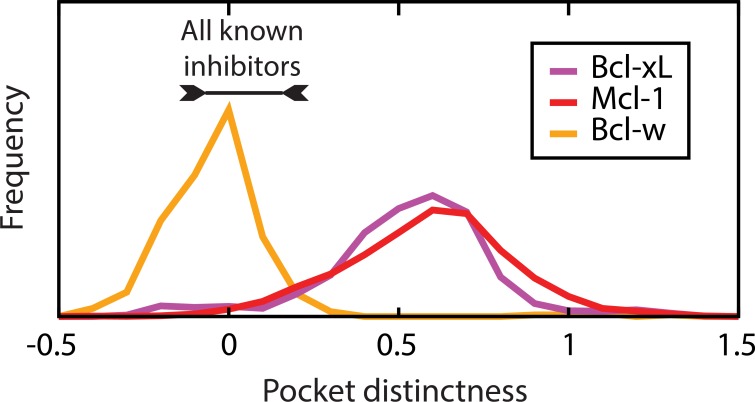
Ensembles of available pocket shapes contain distinct pocket shapes. We define the “distinctness” of a pocket as the difference in exemplar distances of the closest conformation from a different family member, and the closest conformation from one’s own ensemble. Histograms are shown over conformations that comprise the ensembles used above. By this measure, *all* known inhibitors of *all* Bcl-2 family members bind to pockets that are not unique to their cognate target protein (i.e. low “distinctness”). Data are shown for three representative Bcl-2 family members, the complete set are included as **S7 Fig**.

We evaluated the “distinctness” of each conformation in the ensembles presented above, and compiled the results for each family member into a histogram (**Fig. 6**). As expected, these results are consistent with our observations from the MDS analysis presented above: all of the pockets sampled by Bcl-w strongly resemble pockets from other family members and are therefore not “distinct,” whereas Bcl-xL and Mcl-1 each sample certain highly distinct regions. Particularly striking from this analysis is the “distinctness” of experimentally-derived inhibitor-bound structures of Bcl-2 family members: none of these inhibitors take advantage of the “highly distinct” pockets available on the surfaces of Bcl-xL or Mcl-1. Rather, each of these compounds targets a pocket that is sampled not only by the cognate binding partner, but also by at least one other member of the Bcl-2 family.

### Pocket shape similarity as a predictor of ligand selectivity

As noted in the MDS analysis, the majority of known Bcl-xL inhibitors fall in a region of “pocket space” that is sampled by both Bcl-xL and Bcl-2 (**Fig. 5A**). Given that Bcl-2 is found to form pockets that are similar to these inhibitor-bound Bcl-xL pockets, one might expect that these compounds would inhibit Bcl-2 in addition to Bcl-xL. Conversely, in light of the conformational selection model presented earlier, one would expect that the *lack* of similar pockets in the ensembles generated for other Bcl-2 family members might suggest that these proteins would *not* be inhibited by these compounds.

To explore this idea, we measured the exemplar distance between each known inhibitor bound pocket and each pocket observed in a given ensemble (starting from an unbound or peptide-bound structure). We then compared the most similar exemplar distances for each protein/inhibitor pair to experimentally-derived binding data, to evaluate whether the pockets sampled in these ensembles dictate the Bcl-2 family members that will be inhibited by a given compound.

**Fig 7:**
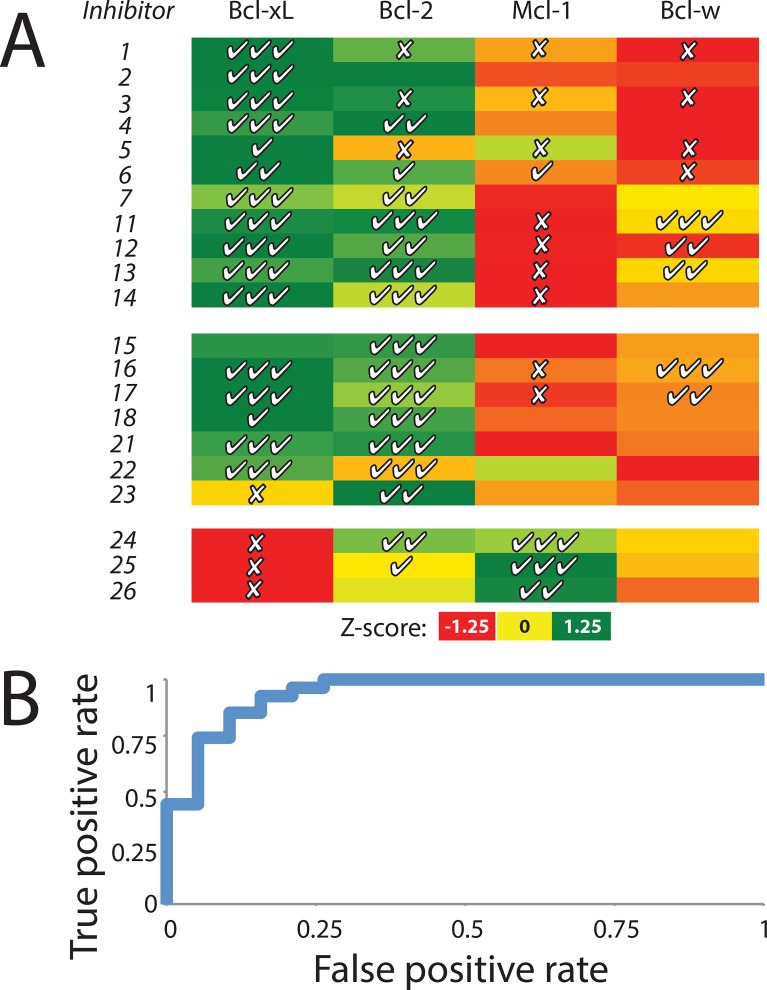
Ensembles of available pocket shapes explain ligand selectivity across the Bcl-2 family. (A) For each inhibitor-bound pocket, the exemplar distance to the most similar pocket is indicated by color gradient, with all distances expressed as Z-scores (*green* are most similar, *red* are most dissimilar; the range of colors for each row is normalized across that row). Markings inside the cells denote experimental reports [11,51,60,67–76] for a given protein-ligand pair (Kd or Ki where available, otherwise IC50): ✔✔✔ indicates < 0.1 μM, ✔✔ indicates 0.1–1 μM, ✔ indicates binding weaker than 1 μM, and ✗ indicates that binding was not detectable/quantifiable. Cells that do not include any markings correspond to protein-ligand pairs for which binding data has not been reported. Numbering corresponds to complexes as in S1 Table. A representative subset of the complexes are included in this figure; a corresponding figure containing all complexes is available as S8 Fig. The underlying raw data are included in S5 Table (exemplar distances) and S7 Table (citations to binding data). (B) A receiver operating characteristic (ROC) plot demonstrating the performance of exemplar distances for predicting whether a given compound is active against a particular member of the Bcl-2 protein family.

The results from this analysis are presented in **Fig. 7A**. Using pockets observed in inhibitor-bound crystal structures of Bcl-xL (***1–14***), we find in many cases that highly similar pockets are sampled in ensembles generated from simulation of Bcl-xL and Bcl-2 (*green*), but not in the corresponding ensembles from Mcl-1 or Bcl-w (*yellow/red*): this represents a quantitative recapitulation of our observation that these ligands occupy surface pockets accessible only to Bcl-xL and Bcl-2 (**Fig. 5A**). In light of this finding such compounds would be expected to bind Bcl-xL and Bcl-2, but not Mcl-1 or Bcl-w; available experimental binding data confirm that indeed this is generally the case.

We observe a similar pattern for most inhibitor-bound crystal structures of Bcl-2 (***15–21***), which is again unsurprising given the similarity of these inhibitor-bound pockets to those of Bcl-xL (**Fig. 2C**). The sole exceptions to this pattern are complexes ***22*** and ***23***, for which corresponding pockets are observed in either the Bcl-2 or Bcl-xL ensembles but not both. The former (***22***) is indeed a dual inhibitor, but binds in a shallow pocket that is not well-described by any Bcl-2 exemplar in our ensemble. The latter (***23***) is indeed selective for Bcl-2 over Bcl-xL, as anticipated from the lack of overlap with the Bcl-xL ensemble from simulation, and therefore represents successful recapitulation of the binding data.

While we noted earlier that Mcl-1 can adopt conformations with highly distinct surface pockets, we also noted that inhibitor-bound crystal structures do not make use of these pockets (**Fig. 6**). Rather, we find that these compounds (***24–26***) instead bind to pockets that are very similar to those included in the Bcl-2 ensemble (**Fig. 7A**), and indeed experimental observations confirm that these compounds also inhibit Bcl-2, but not Bcl-xL.

Among the most notable incorrect predictions are those involving Bcl-w: a number of these compounds inhibit Bcl-w (***11*, *12*, *13*, *16*, *17***), but corresponding pocket shapes were not included in our sampling. In retrospect, this may have arisen due to structural features of the starting conformation from which this conformational ensemble was generated: when evaluated by Molprobity [65], this member of the NMR ensemble contained only 72% of residues in favorable regions of Ramachandran space. These unfavorable structural features may in turn have led to a lack of convergence of our simulations.

Overall, however, there is a striking relationship between the pockets visited by a given ensemble and the experimentally-derived ligand selectivity. To quantitatively examine the ability of the pockets sampled in these ensembles to recapitulate the selectivity profile of a given ligand, we asked how well this approach could be used to distinguish the most tightly binding protein-ligand pairs (those marked with ✔✔✔) from those pairs that bind too weakly to be detected/quantified (those marked with ✗). For each inhibitor-bound crystal structure, we normalized the exemplar distances to the variation across the corresponding row; this essentially expressed ligand selectivity as a Z-score indicating how closely a given ensemble approached the inhibitor-bound pocket. Using these Z-scores to rank the likelihood of interaction for a given protein-ligand pair, we used a receiver operating characteristic (ROC) plot to show performance at this binary prediction problem (**Fig. 7B**); we find that the predictions from this method far outperform those of a random classifier (p < 4x10^-7^).

The absence of experimental binding data for many protein-ligand pairs is a natural shortcoming associated with culling this information from available reports in the literature. The complete maps of ligand selectivity for each compound, as inferred from ensembles generated by simulation, thus stand as completely new predictions in many cases (**S8 Fig.**).

Because the pockets should match the shape of the ligand, a reasonable assumption would be that similar results would be found by comparing the shape of the inhibitor to the ensemble of pocket shapes. To explore this idea we measured the exemplar distance between each native inhibitor conformer and the most similar pocket observed in a given ensemble of Bcl-2 family members, and then compared these exemplar distances to experimentally-derived binding data. As expected, the predictions (**S9 Fig.**) are very similar when comparing to the ligand directly, as opposed to comparing against the ligand-bound pocket.

### Beyond the Bcl-2 family

**Fig 8:**
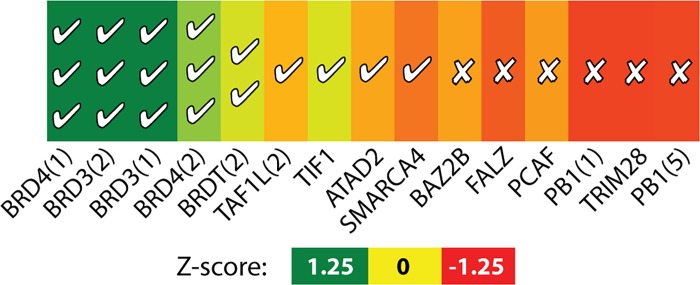
Pocket shapes explain (+)-JQ1 selectivity across bromodomains. For each bromodomain, the exemplar distance of (+)-JQ1 to the most similar pocket is indicated by color gradient, with all distances expressed as Z-scores (*green* are most similar, *red* are most dissimilar; the range of colors for each row is normalized across that row). Markings inside the cells denote experimental binding measurements [10] for each protein-ligand pair: ✔✔✔ indicates ΔTm > 7°C, ✔✔ indicates ΔTm = 3–5°C, ✔ indicates ΔTm = 0–1°C, and ✗ indicates no detectable binding. The underlying raw data are included in **S8 Table**.

The abundance of reported inhibitors of Bcl-2 family members, including their selectivity across the Bcl-2 family, enabled the detailed comparison presented above. Due to the challenges encountered to date in identifying small-molecule inhibitors of protein interactions, however, there do not yet exist any further examples that we know of in which multiple compounds target different members of a protein family. Nonetheless, we were able to identify a separate example of a small-molecule inhibitor of a protein interaction that has been shown to inhibit select members of a protein family (for which at least one ligand-bound crystal structure is available): this is (+)-JQ1, a compound shown to selectively inhibit a subset of human bromodomains [10]. While only one compound, binding data are available against many family members. Here, we have generated ensembles of pocket-containing conformations for 16 bromodomains, and measured the extent to which each family member samples a pocket similar to that observed in the crystal structure of (+)-JQ1 bound to the first bromodomain of BRD4. In this single additional example, here again we find that the presence of complementary surface pockets in the ensemble generated by simulation can accurately predict the ligand selectivity across a protein family (**Fig. 8**), whereas we do not observe complementary pockets for family members that do not tightly bind (+)-JQ1.

## Discussion

Identification of small-molecule inhibitors of protein interactions immediately raised the question of how these compounds might interact with proteins that appeared to lack complementary surface pockets; the answer came through structural studies of an interleukin-2 complex that showed the ligand can occupy a hydrophobic groove not present on the unbound protein surface [13]. These structural studies, together with analysis of binding thermodynamics, first pointed to the “adaptivity” of this protein surface: the protein can adopt multiple conformations, one of which presents a surface complementary to the ligand. Our analysis of Bcl-2 family complexes supports this view, and extends it further: we find that the plasticity of the protein surface allows multiple distinct surface pockets to be presented, and these different pockets can be recognized by inhibitors with dramatically different chemotypes.

Upon generating ensembles of pocket-containing conformations by simulation, we find that these ensembles span all the pockets used by known inhibitors—even though no information about any inhibitor was used to influence the simulations in any way. This observation provides strong and direct evidence for an underlying model of conformational selection [15]: the protein surface is predisposed to adopt certain pocket shapes, and these shapes in turn restrict the range of complementary ligands. Upon binding, the protein may then undergo further smaller changes in response to particular features of the ligand.

By comparing the regions of “pocket space” explored by several Bcl-2 family members, we find that certain shapes are available to all family members: we expect that compounds complementing such pockets will show very broad specificity across this family. We further find that most inhibitors reported in the literature bind to pockets that are shared by more than one family member, and accordingly most are found to be active against more than one family member.

Conversely, we also find that many Bcl-2 family members sample pockets that are not accessible to any other family member: here lies a tremendous opportunity, since we expect that a compound built to complement such a pocket will prove highly selective for its target. Development of compounds that target these highly “distinct” pockets represents a tantalizing new strategy for drug discovery: by building target selectivity into the broad features of the chemical scaffold itself, selectivity may be more robustly preserved in the course of optimization of the compound for other orthogonal desirable properties (bioavailability, pharmacokinetics and pharmacology). Despite the existence of these pockets on the surface of Bcl-2 family members and extensive interest in identifying selective inhibitors, however, not a single crystal structure reported to date includes a compound that targets any of these highly “distinct” pockets.

How then can we identify compounds that achieve target selectivity by explicitly targeting these “distinct” pockets? We anticipate the solution may lie with the exemplars themselves. As noted earlier, the exemplar is essentially a map of the “perfect” ligand to complement a given pocket, albeit a ligand that is not physically realizable. Accordingly, we expect that the exemplar will serve as an ideal template for ligand-based screening of (virtual) compound libraries; tools such as ROCS [39,40] that evaluate volume and chemical overlap may be used to find compounds that closely mimic the shape and chemical features of the exemplar. Indeed, the ability to assess the selectivity profile by comparing the native ligand conformer to the exemplars derived from the ensembles implies that ROCS is capable of identifying compounds with the desired shape and chemical features needed to strongly interact with a member of a protein’s ensemble. Together, this set of tools may provide both a means to identify pockets that “encode” a desired selectivity profile within a protein family, and also a means to connect the resulting pockets to specific compounds that exhibit this selectivity profile.

The overall paucity of examples of small-molecule inhibitors of protein interactions necessitated our focus for this study be largely restricted to the Bcl-2 family. As selective inhibitors of other protein families involved in protein interactions are reported, it will be exciting to refine the insights presented here. In light of the fact that many of the other small-molecule inhibitors of protein interactions described to date also bind to similarly “adaptable” binding sites [66], meanwhile, we are optimistic that the perspectives presented here will prove extensible to these therapeutic targets as well.

## Supporting Information

S1 Fig Effect of orientation on exemplar comparison. Bcl-xL bound to ABT-737 (PDB ID 2yxj), a compound similar to ABT-737 (PDB ID 3qkd), an unrelated Bcl-xL inhibitor (PDB ID 3zln), and Mcl-1 bound to an Mcl-1 inhibitor (PDB ID 4hw2) were rotated for 100 random orientations, and an all-vs-all comparison of the ensemble of ABT-737 bound exemplars to those generated for each orientation was performed. Histograms of the 3D Tanimoto scores of the ABT-737 exemplars compared to themselves (*black*), a closely-related compound (*green*), an unrelated compound (*orange*), and an Mcl-1 pocket (*red*) demonstrate overlap between similar and dissimilar pockets.
(EPS)

S2 Fig Bcl-2 family members recognize different inhibitors using distinct surface pockets. In all cases color gradient indicates similarity between experimentally-derived structures, expressed as Z-scores (*green* are most similar, *red* are most dissimilar). Numbering in all cases corresponds to complexes in **S1 Table**. (A) Chemical similarity of the inhibitors. (B) Three-dimensional similarity of the inhibitors’ active conformation. (C) Similarity of protein surface pockets, quantified through exemplar similarity. There is a statistically significant correlation between each pair of panels, as described in the text. The underlying raw data used to generate these plots are included in **S2, S3, and S4 Tables**.
(EPS)

S3 Fig Steric clashes with ground state conformations demonstrate the need to explore ensembles of pocket-containing conformations to understand ligand binding. Unbound structures of (A) Bcl-xL, (B) Bcl-2, and (C) Mcl-1 are represented in spheres with overlaid inhibitors shown in sticks.
(EPS)

S4 Fig Location of target residues. A superposition of representative inhibitor-bound structures of Bcl-xL (*light green cartoon*, *inhibitor in dark green sticks*) and Mcl-1 (*cyan cartoon*, *inhibitor in dark blue sticks*) is shown. The target residues for pocket opening generated from the corresponding peptide-bound structures differed for Bcl-xL (Ala93 and Arg143) and for Mcl-1 (Arg263 and Phe270) (*sidechains shown using spacefill*), reflecting different energetic contributions to the protein-protein interaction from each protein surface.
(EPS)

S5 Fig Energetic analysis of conformations generated starting from a peptide bound structure. For each peptide-bound complex used in this analysis, the histograms of energies of conformations generated without the use of a biasing potential (*black line*) overlap with the range of energies of the conformations used in all subsequent analyses generated by using a biasing potential at the Bcl-xL protein interaction site (*magenta line*) and the Mcl-1 protein interaction site (*red line*); this suggests that many of these conformations are energetically accessible to these proteins under physiological conditions. All energies shown here were evaluated in the absence of the biasing potential, for fair comparison.
(PNG)

S6 Fig Comparison of pocket space for human and mouse Mcl-1. Constructs of Mcl-1 were made from peptide-bound (2nl9 and 3mk8) and unbound (1wsx) structures of Mcl-1 with human (*red*) and mouse (*green*) sequences. Individual conformations are represented as points on a two-dimensional projection that best reflects the pairwise distances between their exemplars.
(EPS)

S7 Fig “Distinctness” of pockets in ensembles generated by simulation of Bcl-2 family members. The “distinctness” of a pocket is defined as the exemplar distance of the difference in exemplar distances of the closest conformation from a different family member, and the closest conformation from the ensemble of this family member.
(EPS)

S8 Fig Pocket shape similarity explains ligand selectivity across the Bcl-2 family. For each inhibitor-bound pocket, the exemplar distance to the most similar pocket is indicated by color gradient expressed as Z-scores (*green* are most similar, *red* are most dissimilar; the range of colors for each row is normalized across that row). Markings inside the cells denote experimental measurements of binding for this protein-ligand pair (Kd or Ki where available, otherwise IC50): ✔✔✔ indicates < 0.1 μM, ✔✔ indicates 0.1–1 μM, ✔ indicates binding weaker than 1 μM, and ✗ indicates that binding was not detectable/quantifiable. Cells that do not include markings correspond to protein-ligand pairs for which binding data has not been reported. Numbering corresponds to complexes as in **S1 Table**. The underlying raw data are included in **S5 Table** (exemplar distances) and **S7 Table** (citations to binding data).
(EPS)

S9 Fig Pocket shape versus ligand shape similarity explains ligand selectivity across the Bcl-2 family. As opposed to S8 Fig. , in which we compared conformations from simulation to exemplars from inhibitor-bound pockets, here we compare conformations from simulation directly to the structures of the inhibitors. As previously, the exemplar distance to the most similar pocket is indicated by color gradient expressed as Z-scores (*green* are most similar, *red* are most dissimilar; the range of colors for each row is normalized across that row). Markings inside the cells denote experimental measurements of binding for this protein-ligand pair (Kd or Ki where available, otherwise IC50): ✔✔✔ indicates < 0.1 μM, ✔✔ indicates 0.1–1 μM, ✔ indicates binding weaker than 1 μM, and ✗ indicates that binding was not detectable/quantifiable. Cells that do not include markings correspond to protein-ligand pairs for which binding data has not been reported. Numbering corresponds to complexes as in **S1 Table**. The underlying raw data are included in **S6 Table** (exemplar distances) and **S7 Table** (citations to binding data).
(EPS)

S1 Table Structures of complexes used in this study. At this time of writing, this table represents a comprehensive collection of all structures in the PDB containing a Bcl-2 family member in complex with a small-molecule inhibitor bound at the protein interaction site. Fragments (compounds with molecular weight less than 250 Da), molecules whose structure contains interactions with multiple chains that are not part of a biological unit, and molecules with multiple occupancies were excluded from this list. Numbering of compounds in this list corresponds to the order of rows in Fig. 2 and Fig. 7, and the numbering of compounds in Fig. 4 and Fig. 5A. Superscripted letters in the leftmost column denote cases in which the same compound has been solved in complex with different protein partners.
(DOCX)

S2 Table 2D Tanimoto similarity of Bcl-2 family inhibitors. This table shows the raw data from which the heatmap in Fig. 2A was created.
(DOCX)

S3 Table 3D Tanimoto (shape) similarity of Bcl-2 family inhibitors. This table shows the raw data from which the heatmap in Fig. 2B was created.
(DOCX)

S4 Table Exemplar similarity of Bcl-2 family inhibitor bound structures. This table shows the raw data from which the heatmap in Fig. 2C was created.
(DOCX)

S5 Table Exemplar similarity of top (closest) pocket optimized structures to inhibitor-bound structures. This table shows the raw data from which Fig. 7 and S8 Fig. were created.
(DOCX)

S6 Table Exemplar similarity of top (closest) pocket optimized structures to native inhibitor conformer. This table shows the raw data from which S9 Fig. was created.
(DOCX)

S7 Table Citations for experimental data for ligand selectivity. This table points to the sources of experimental data from which Figs. 7, S8, and S9 were created. Citations are as follows: *A*: Lessene G, Czabotar PE, Sleebs BE, Zobel K, Lowes KN, et al. (2013) Structure-guided design of a selective BCL-X(L) inhibitor. Nat Chem Biol 9: 390–397. *B*: Schroeder GM, Wei D, Banfi P, Cai ZW, Lippy J, et al. (2012) Pyrazole and pyrimidine phenylacylsulfonamides as dual Bcl-2/Bcl-xL antagonists. Bioorg Med Chem Lett 22: 3951–3956. *C*: Brady RM, Vom A, Roy MJ, Toovey N, Smith BJ, et al. (2014) De-novo designed library of benzoylureas as inhibitors of BCL-XL: synthesis, structural and biochemical characterization. J Med Chem 57: 1323–1343. *D*: Oltersdorf T, Elmore SW, Shoemaker AR, Armstrong RC, Augeri DJ, et al. (2005) An inhibitor of Bcl-2 family proteins induces regression of solid tumours. Nature 435: 677–681. *E*: Lee EF, Czabotar PE, Yang H, Sleebs BE, Lessene G, et al. (2009) Conformational changes in Bcl-2 pro-survival proteins determine their capacity to bind ligands. J Biol Chem 284: 30508–30517. *F*: Sleebs BE, Czabotar PE, Fairbrother WJ, Fairlie WD, Flygare JA, et al. (2011) Quinazoline sulfonamides as dual binders of the proteins B-cell lymphoma 2 and B-cell lymphoma extra long with potent proapoptotic cell-based activity. J Med Chem 54: 1914–1926. *G*: Zhou H, Aguilar A, Chen J, Bai L, Liu L, et al. (2012) Structure-based design of potent Bcl-2/Bcl-xL inhibitors with strong in vivo antitumor activity. J Med Chem 55: 6149–6161. *H*: Touré BB, Miller-Moslin K, Yusuff N, Perez L, Doré M, et al. (2013) The Role of the Acidity of N-Heteroaryl Sulfonamides as Inhibitors of Bcl-2 Family Protein–Protein Interactions. ACS Med Chem Lett 4: 186–190. *I*: Petros AM, Dinges J, Augeri DJ, Baumeister SA, Betebenner DA, et al. (2006) Discovery of a potent inhibitor of the antiapoptotic protein Bcl-xL from NMR and parallel synthesis. J Med Chem 49: 656–663. *J*: Bruncko M, Oost TK, Belli BA, Ding H, Joseph MK, et al. (2007) Studies leading to potent, dual inhibitors of Bcl-2 and Bcl-xL. J Med Chem 50: 641–662. *K*: Perez HL, Banfi P, Bertrand J, Cai ZW, Grebinski JW, et al. (2012) Identification of a phenylacylsulfonamide series of dual Bcl-2/Bcl-xL antagonists. Bioorg Med Chem Lett 22: 3946–3950. *L*: Porter J, Payne A, de Candole B, Ford D, Hutchinson B, et al. (2009) Tetrahydroisoquinoline amide substituted phenyl pyrazoles as selective Bcl-2 inhibitors. Bioorg Med Chem Lett 19: 230–233. *M*: Friberg A, Vigil D, Zhao B, Daniels RN, Burke JP, et al. (2013) Discovery of potent myeloid cell leukemia 1 (Mcl-1) inhibitors using fragment-based methods and structure-based design. J Med Chem 56: 15–30. *N*: Tanaka Y, Aikawa K, Nishida G, Homma M, Sogabe S, et al. (2013) Discovery of potent Mcl-1/Bcl-xL dual inhibitors by using a hybridization strategy based on structural analysis of target proteins. J Med Chem 56: 9635–9645.
(DOCX)

S8 Table Exemplar similarity of top (closest) pocket optimized structures to (+)-JQ1-BRD4(1) bound structure. This table shows the raw data from which the heatmap in Fig. 8 was created.
(DOCX)

S1 Text Supplementary methods. This supporting text contains a complete description of methodology used, including PDB structures used in calculations. A description of pocket-opened conformations for each protein in our test set is also included in this text.
(DOCX)
