# Supplementary figures and images for: Selectivity by Small-Molecule Inhibitors of Protein Interactions Can Be Driven by Protein Surface Fluctuations
Source: PLoS Comput Biol. 2015 Feb 23;11(2):e1004081. doi: 10.1371/journal.pcbi.1004081 (PMC4338137; doi:10.1371/journal.pcbi.1004081)

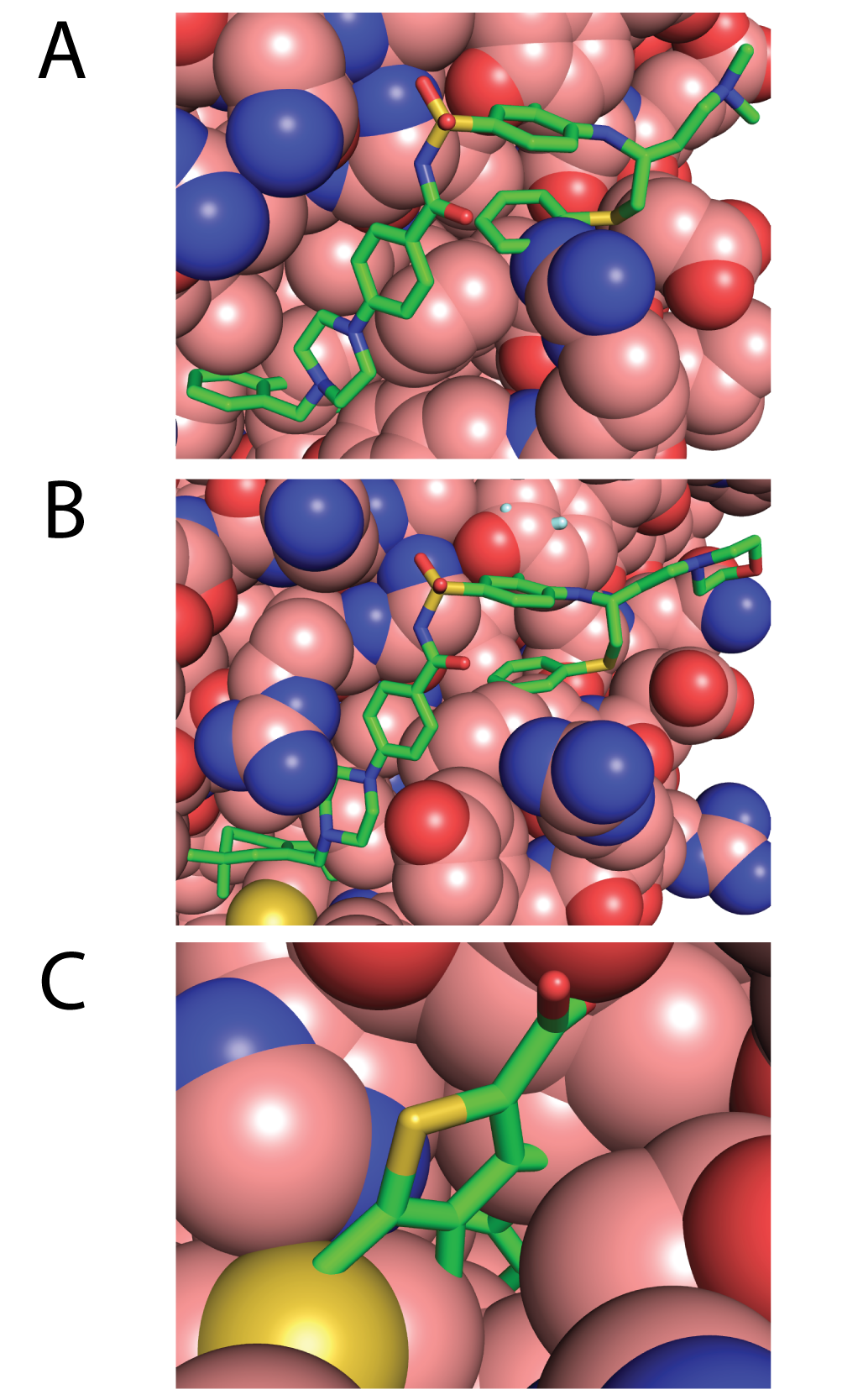

Supplement: S5 Fig — For each peptide-bound complex used in this analysis, the histograms of energies of conformations generated without the use of a biasing potential (black line) overlap with the range of energies of the conformations used in all subsequent analyses generated by using a biasing potential at the Bcl-xL protein interaction site (magenta line) and the Mcl-1 protein interaction site (red line); this suggests that many of these conformations are energetically accessible to these proteins under physiological conditions. All energies shown here were evaluated in the absence of the biasing potential, for fair comparison. (PNG) [file pcbi.1004081.s005.png]
