# Supplementary material for: Selectivity by Small-Molecule Inhibitors of Protein Interactions Can Be Driven by Protein Surface Fluctuations
Source: PLoS Comput Biol. 2015 Feb 23;11(2):e1004081. doi: 10.1371/journal.pcbi.1004081 (PMC4338137; doi:10.1371/journal.pcbi.1004081)
Supplement: S1 Table — At this time of writing, this table represents a comprehensive collection of all structures in the PDB containing a Bcl-2 family member in complex with a small-molecule inhibitor bound at the protein interaction site. Fragments (compounds with molecular weight less than 250 Da), molecules whose structure contains interactions with multiple chains that are not part of a biological unit, and molecules with multiple occupancies were excluded from this list. Numbering of compounds in this list corresponds to the order of rows in Fig. 2 and Fig. 7, and the numbering of compounds in Fig. 4 and Fig. 5A. Superscripted letters in the leftmost column denote cases in which the same compound has been solved in complex with different protein partners. (DOCX) [file pcbi.1004081.s010.docx]

| Complex | PDB ID | Protein | Inhibitor | Inhibitor  common name |
| --- | --- | --- | --- | --- |
| *1* | 3zln | Bcl‑xL |  |  |
| *2* | 3zlo | Bcl‑xL |  |  |
| *3* | 3zlr | Bcl‑xL |  | WEHI‑539 |
| *4* | 4ehr | Bcl‑xL |  |  |
| *5* | 4c52 | Bcl‑xL |  |  |
| *6* | 4c5d | Bcl‑xL |  |  |
| *7* | 1ysi | Bcl‑xL |  |  |
| *8 ^a^* | 1ysn | Bcl‑xL |  |  |
| *9 ^b^* | 2o2m | Bcl‑xL |  |  |
| *10* | 2o2n | Bcl‑xL |  |  |
| *11* | 2yxj | Bcl‑xL |  | ABT-737 |
| *12* | 3inq | Bcl‑xL |  | W1191542 |
| *13* | 3qkd | Bcl‑xL |  |  |
| *14* | 3sp7 | Bcl‑xL |  | BM-903 |
| *15* | 4ieh | Bcl‑2 |  |  |
| *16* | 4lvt | Bcl‑2 |  | ABT‑263 (navitoclax) |
| *17* | 4man | Bcl‑2 |  | ABT-199 |
| *18* | 4lxd | Bcl‑2 |  |  |
| *19 ^b^* | 2o2f | Bcl‑2 |  |  |
| *20 ^a^* | 1ysw | Bcl‑2 |  |  |
| *21* | 2o22 | Bcl‑2 |  |  |
| *22* | 4aq3 | Bcl‑2 |  |  |
| *23* | 2w3l | Bcl‑2 |  |  |
| *24* | 4hw2 | Mcl‑1 |  |  |
| *25* | 4hw3 | Mcl‑1 |  |  |
| *26* | 3wix | Mcl‑1 |  |  |
| *27* | 4oq5 | Mcl‑1 |  |  |
| *28* | 4oq6 | Mcl‑1 |  |  |

Table S1: Structures of complexes used in this study. At this time of writing, this table represents a comprehensive collection of all structures in the PDB containing a Bcl‑2 family member in complex with a small-molecule inhibitor bound at the protein interaction site. Fragments (compounds with molecular weight less than 250 Da), molecules whose structure contains interactions with multiple chains that are not part of a biological unit, and molecules with multiple occupancies were excluded from this list. Numbering of compounds in this list corresponds to the order of rows in **Figure 2** and **Figure 7**, and the numbering of compounds in **Figure 4** and **Figure 5a**. Superscripted letters in the leftmost column denote cases in which the same compound has been solved in complex with different protein partners.
