# Supplementary material for: Selectivity by Small-Molecule Inhibitors of Protein Interactions Can Be Driven by Protein Surface Fluctuations
Source: PLoS Comput Biol. 2015 Feb 23;11(2):e1004081. doi: 10.1371/journal.pcbi.1004081 (PMC4338137; doi:10.1371/journal.pcbi.1004081)
Supplement: S2 Table — This table shows the raw data from which the heatmap in Fig. 2A was created. (DOCX) [file pcbi.1004081.s011.docx]

Table S2: 2D Tanimoto similarity of Bcl‑2 family inhibitors. This table shows the raw data from which the heatmap in Figure 2a was created.
