# Supplementary material for: Selectivity by Small-Molecule Inhibitors of Protein Interactions Can Be Driven by Protein Surface Fluctuations
Source: PLoS Comput Biol. 2015 Feb 23;11(2):e1004081. doi: 10.1371/journal.pcbi.1004081 (PMC4338137; doi:10.1371/journal.pcbi.1004081)
Supplement: S5 Table — This table shows the raw data from which Fig. 7 and S8 Fig. were created. (DOCX) [file pcbi.1004081.s014.docx]

| **Complex** | **Bcl‑xL** | **Bcl‑2** | **Mcl‑1** | **Bcl‑w** | **Bax** | **Bid** | **Ced‑9** |
| --- | --- | --- | --- | --- | --- | --- | --- |
| ***1*** | 0.58 | 0.55 | 0.49 | 0.45 | 0.55 | 0.48 | 0.51 |
| ***2*** | 0.75 | 0.74 | 0.52 | 0.51 | 0.58 | 0.55 | 0.59 |
| ***3*** | 0.65 | 0.64 | 0.52 | 0.41 | 0.51 | 0.55 | 0.57 |
| ***4*** | 0.67 | 0.72 | 0.51 | 0.42 | 0.53 | 0.52 | 0.60 |
| ***5*** | 0.88 | 0.68 | 0.75 | 0.56 | 0.66 | 0.71 | 0.79 |
| ***6*** | 0.85 | 0.70 | 0.51 | 0.45 | 0.49 | 0.56 | 0.54 |
| ***7*** | 0.67 | 0.64 | 0.54 | 0.61 | 0.71 | 0.67 | 0.51 |
| ***8*** | 0.65 | 0.59 | 0.54 | 0.49 | 0.58 | 0.56 | 0.63 |
| ***9*** | 0.61 | 0.60 | 0.47 | 0.50 | 0.63 | 0.58 | 0.54 |
| ***10*** | 0.67 | 0.69 | 0.43 | 0.55 | 0.58 | 0.57 | 0.46 |
| ***11*** | 0.71 | 0.72 | 0.44 | 0.57 | 0.62 | 0.61 | 0.45 |
| ***12*** | 0.81 | 0.73 | 0.53 | 0.54 | 0.61 | 0.62 | 0.66 |
| ***13*** | 0.69 | 0.72 | 0.43 | 0.56 | 0.66 | 0.59 | 0.44 |
| ***14*** | 0.70 | 0.60 | 0.48 | 0.55 | 0.61 | 0.61 | 0.52 |
| ***15*** | 0.74 | 0.74 | 0.47 | 0.57 | 0.68 | 0.68 | 0.48 |
| ***16*** | 0.70 | 0.65 | 0.57 | 0.58 | 0.62 | 0.59 | 0.54 |
| ***17*** | 0.87 | 0.73 | 0.54 | 0.59 | 0.71 | 0.70 | 0.51 |
| ***18*** | 0.78 | 0.74 | 0.57 | 0.59 | 0.70 | 0.65 | 0.50 |
| ***19*** | 0.76 | 0.78 | 0.52 | 0.66 | 0.74 | 0.71 | 0.56 |
| ***20*** | 0.58 | 0.63 | 0.46 | 0.54 | 0.63 | 0.63 | 0.54 |
| ***21*** | 0.59 | 0.59 | 0.49 | 0.51 | 0.57 | 0.57 | 0.49 |
| ***22*** | 0.61 | 0.53 | 0.57 | 0.42 | 0.59 | 0.59 | 0.56 |
| ***23*** | 0.67 | 0.77 | 0.66 | 0.64 | 0.64 | 0.64 | 0.77 |
| ***24*** | 0.47 | 0.63 | 0.62 | 0.58 | 0.59 | 0.61 | 0.64 |
| ***25*** | 0.48 | 0.63 | 0.78 | 0.61 | 0.69 | 0.66 | 0.61 |
| ***26*** | 0.50 | 0.71 | 0.83 | 0.61 | 0.79 | 0.75 | 0.68 |
| ***27*** | 0.74 | 0.90 | 0.98 | 0.91 | 1.11 | 1.04 | 0.93 |
| ***28*** | 0.61 | 0.76 | 0.84 | 0.71 | 1.12 | 0.87 | 0.81 |

Table S5: Exemplar similarity of top (closest) pocket optimized structures to inhibitor-bound structures. This table shows the raw data from which Figure 7 and Figure S8 were created.
