# Supplementary material for: Selectivity by Small-Molecule Inhibitors of Protein Interactions Can Be Driven by Protein Surface Fluctuations
Source: PLoS Comput Biol. 2015 Feb 23;11(2):e1004081. doi: 10.1371/journal.pcbi.1004081 (PMC4338137; doi:10.1371/journal.pcbi.1004081)
Supplement: S6 Table — This table shows the raw data from which S9 Fig. was created. (DOCX) [file pcbi.1004081.s015.docx]

| **Complex** | **Bcl‑xL** | **Bcl‑2** | **Mcl‑1** | **Bcl‑w** | **Bax** | **Bid** | **Ced‑9** |
| --- | --- | --- | --- | --- | --- | --- | --- |
| ***1*** | 0.65 | 0.65 | 0.69 | 0.51 | 0.67 | 0.65 | 0.60 |
| ***2*** | 0.68 | 0.52 | 0.50 | 0.47 | 0.56 | 0.64 | 0.54 |
| ***3*** | 0.60 | 0.54 | 0.47 | 0.47 | 0.54 | 0.62 | 0.53 |
| ***4*** | 0.52 | 0.46 | 0.46 | 0.48 | 0.49 | 0.52 | 0.40 |
| ***5*** | 0.65 | 0.63 | 0.51 | 0.52 | 0.56 | 0.61 | 0.63 |
| ***6*** | 0.60 | 0.52 | 0.50 | 0.51 | 0.53 | 0.57 | 0.51 |
| ***7*** | 0.64 | 0.53 | 0.52 | 0.50 | 0.54 | 0.57 | 0.49 |
| ***8*** | 0.54 | 0.49 | 0.43 | 0.48 | 0.51 | 0.50 | 0.45 |
| ***9*** | 0.59 | 0.56 | 0.45 | 0.51 | 0.53 | 0.54 | 0.45 |
| ***10*** | 0.53 | 0.52 | 0.43 | 0.51 | 0.50 | 0.56 | 0.40 |
| ***11*** | 0.52 | 0.45 | 0.42 | 0.47 | 0.44 | 0.50 | 0.38 |
| ***12*** | 0.52 | 0.45 | 0.42 | 0.44 | 0.46 | 0.49 | 0.40 |
| ***13*** | 0.51 | 0.46 | 0.41 | 0.49 | 0.45 | 0.48 | 0.37 |
| ***14*** | 0.49 | 0.46 | 0.41 | 0.46 | 0.43 | 0.46 | 0.32 |
| ***15*** | 0.52 | 0.44 | 0.42 | 0.49 | 0.45 | 0.49 | 0.37 |
| ***16*** | 0.48 | 0.43 | 0.43 | 0.48 | 0.39 | 0.46 | 0.37 |
| ***17*** | 0.52 | 0.50 | 0.44 | 0.53 | 0.44 | 0.48 | 0.39 |
| ***18*** | 0.52 | 0.51 | 0.44 | 0.47 | 0.45 | 0.47 | 0.41 |
| ***19*** | 0.61 | 0.58 | 0.47 | 0.53 | 0.54 | 0.55 | 0.45 |
| ***20*** | 0.53 | 0.49 | 0.41 | 0.50 | 0.47 | 0.51 | 0.42 |
| ***21*** | 0.64 | 0.61 | 0.50 | 0.55 | 0.59 | 0.58 | 0.52 |
| ***22*** | 0.58 | 0.52 | 0.45 | 0.54 | 0.52 | 0.57 | 0.48 |
| ***23*** | 0.46 | 0.52 | 0.46 | 0.46 | 0.59 | 0.60 | 0.43 |
| ***24*** | 0.60 | 0.65 | 0.69 | 0.65 | 0.66 | 0.67 | 0.55 |
| ***25*** | 0.57 | 0.63 | 0.68 | 0.66 | 0.66 | 0.66 | 0.58 |
| ***26*** | 0.54 | 0.58 | 0.62 | 0.58 | 0.59 | 0.58 | 0.43 |
| ***27*** | 0.52 | 0.57 | 0.57 | 0.50 | 0.62 | 0.57 | 0.40 |
| ***28*** | 0.62 | 0.87 | 0.82 | 0.78 | 0.83 | 0.85 | 0.79 |

Table S6: Exemplar similarity of top (closest) pocket optimized structures to native inhibitor conformer. This table shows the raw data from which Figure S9 was created.
