# Supplementary material for: Selectivity by Small-Molecule Inhibitors of Protein Interactions Can Be Driven by Protein Surface Fluctuations
Source: PLoS Comput Biol. 2015 Feb 23;11(2):e1004081. doi: 10.1371/journal.pcbi.1004081 (PMC4338137; doi:10.1371/journal.pcbi.1004081)
Supplement: S7 Table — This table points to the sources of experimental data from which Figs. 7, S8, and S9 were created. Citations are as follows: A: Lessene G, Czabotar PE, Sleebs BE, Zobel K, Lowes KN, et al. (2013) Structure-guided design of a selective BCL-X(L) inhibitor. Nat Chem Biol 9: 390–397. B: Schroeder GM, Wei D, Banfi P, Cai ZW, Lippy J, et al. (2012) Pyrazole and pyrimidine phenylacylsulfonamides as dual Bcl-2/Bcl-xL antagonists. Bioorg Med Chem Lett 22: 3951–3956. C: Brady RM, Vom A, Roy MJ, Toovey N, Smith BJ, et al. (2014) De-novo designed library of benzoylureas as inhibitors of BCL-XL: synthesis, structural and biochemical characterization. J Med Chem 57: 1323–1343. D: Oltersdorf T, Elmore SW, Shoemaker AR, Armstrong RC, Augeri DJ, et al. (2005) An inhibitor of Bcl-2 family proteins induces regression of solid tumours. Nature 435: 677–681. E: Lee EF, Czabotar PE, Yang H, Sleebs BE, Lessene G, et al. (2009) Conformational changes in Bcl-2 pro-survival proteins determine their capacity to bind ligands. J Biol Chem 284: 30508–30517. F: Sleebs BE, Czabotar PE, Fairbrother WJ, Fairlie WD, Flygare JA, et al. (2011) Quinazoline sulfonamides as dual binders of the proteins B-cell lymphoma 2 and B-cell lymphoma extra long with potent proapoptotic cell-based activity. J Med Chem 54: 1914–1926. G: Zhou H, Aguilar A, Chen J, Bai L, Liu L, et al. (2012) Structure-based design of potent Bcl-2/Bcl-xL inhibitors with strong in vivo antitumor activity. J Med Chem 55: 6149–6161. H: Touré BB, Miller-Moslin K, Yusuff N, Perez L, Doré M, et al. (2013) The Role of the Acidity of N-Heteroaryl Sulfonamides as Inhibitors of Bcl-2 Family Protein–Protein Interactions. ACS Med Chem Lett 4: 186–190. I: Petros AM, Dinges J, Augeri DJ, Baumeister SA, Betebenner DA, et al. (2006) Discovery of a potent inhibitor of the antiapoptotic protein Bcl-xL from NMR and parallel synthesis. J Med Chem 49: 656–663. J: Bruncko M, Oost TK, Belli BA, Ding H, Joseph MK, et al. (2007) Studies leading to potent, dual i [file pcbi.1004081.s016.docx]

| **Compound** | **Bcl‑xL** | **Bcl‑2** | **Mcl‑1** | **Bcl‑w** | **Bax** | **Bid** | **Ced‑9** |
| --- | --- | --- | --- | --- | --- | --- | --- |
| ***1*** | *A* | *A* | *A* | *A* |  |  |  |
| ***2*** | *A* |  |  |  |  |  |  |
| ***3*** | *A* | *A* | *A* | *A* |  |  |  |
| ***4*** | *B* | *B* |  |  |  |  |  |
| ***5*** | *C* | *C* | *C* | *C* |  |  |  |
| ***6*** | *C* | *C* | *C* | *C* |  |  |  |
| ***7*** | *D* | *D* |  |  |  |  |  |
| ***8*** |  |  |  |  |  |  |  |
| ***9*** |  |  |  |  |  |  |  |
| ***10*** |  |  |  |  |  |  |  |
| ***11*** | *D* | *D* | *D* | *D* |  |  |  |
| ***12*** | *E* | *E* | *E* | *E* |  |  |  |
| ***13*** | *F* | *F* | *F* | *F* | *S* |  |  |
| ***14*** | *G* | *G* | *G* |  |  |  |  |
| ***15*** |  | *H* |  |  |  |  |  |
| ***16*** | *I* | *I* | *I* | *I* |  |  |  |
| ***17*** | *I* | *I* | *I* | *I* |  |  |  |
| ***18*** | *I* | *I* |  |  |  |  |  |
| ***19*** |  |  |  |  |  |  |  |
| ***20*** |  |  |  |  |  |  |  |
| ***21*** | *J* | *J* |  |  |  |  |  |
| ***22*** | *K* | *K* |  |  |  |  |  |
| ***23*** | *L* | *L* |  |  |  |  |  |
| ***24*** | *M* | *M* | *M* |  |  |  |  |
| ***25*** | *M* | *M* | *M* |  |  |  |  |
| ***26*** | *N* |  | *N* |  |  |  |  |
| ***27*** |  |  |  |  |  |  |  |
| ***28*** |  |  |  |  |  |  |  |

Table S7: Citations for experimental data for ligand selectivity. This table points to the sources of experimental data from which Figures 7, S8, and S9 were created. Citations are as follows:

*A:* Lessene G, Czabotar PE, Sleebs BE, Zobel K, Lowes KN, et al. (2013) Structure-guided design of a selective BCL-X(L) inhibitor. Nat Chem Biol 9: 390-397.

*B:* Schroeder GM, Wei D, Banfi P, Cai ZW, Lippy J, et al. (2012) Pyrazole and pyrimidine phenylacylsulfonamides as dual Bcl-2/Bcl-xL antagonists. Bioorg Med Chem Lett 22: 3951-3956.

*C:* Brady RM, Vom A, Roy MJ, Toovey N, Smith BJ, et al. (2014) De-novo designed library of benzoylureas as inhibitors of BCL-XL: synthesis, structural and biochemical characterization. J Med Chem 57: 1323-1343.

*D:* Oltersdorf T, Elmore SW, Shoemaker AR, Armstrong RC, Augeri DJ, et al. (2005) An inhibitor of Bcl-2 family proteins induces regression of solid tumours. Nature 435: 677-681.

*E:* Lee EF, Czabotar PE, Yang H, Sleebs BE, Lessene G, et al. (2009) Conformational changes in Bcl-2 pro-survival proteins determine their capacity to bind ligands. J Biol Chem 284: 30508-30517.

*F:* Sleebs BE, Czabotar PE, Fairbrother WJ, Fairlie WD, Flygare JA, et al. (2011) Quinazoline sulfonamides as dual binders of the proteins B-cell lymphoma 2 and B-cell lymphoma extra long with potent proapoptotic cell-based activity. J Med Chem 54: 1914-1926.

*G:* Zhou H, Aguilar A, Chen J, Bai L, Liu L, et al. (2012) Structure-based design of potent Bcl-2/Bcl-xL inhibitors with strong in vivo antitumor activity. J Med Chem 55: 6149-6161.

*H:* Touré BB, Miller-Moslin K, Yusuff N, Perez L, Doré M, et al. (2013) The Role of the Acidity of N-Heteroaryl Sulfonamides as Inhibitors of Bcl-2 Family Protein–Protein Interactions. ACS Med Chem Lett 4: 186-190.

*I:* Petros AM, Dinges J, Augeri DJ, Baumeister SA, Betebenner DA, et al. (2006) Discovery of a potent inhibitor of the antiapoptotic protein Bcl-xL from NMR and parallel synthesis. J Med Chem 49: 656-663.

*J:* Bruncko M, Oost TK, Belli BA, Ding H, Joseph MK, et al. (2007) Studies leading to potent, dual inhibitors of Bcl-2 and Bcl-xL. J Med Chem 50: 641-662.

*K:* Perez HL, Banfi P, Bertrand J, Cai ZW, Grebinski JW, et al. (2012) Identification of a phenylacylsulfonamide series of dual Bcl-2/Bcl-xL antagonists. Bioorg Med Chem Lett 22: 3946-3950.

*L:* Porter J, Payne A, de Candole B, Ford D, Hutchinson B, et al. (2009) Tetrahydroisoquinoline amide substituted phenyl pyrazoles as selective Bcl-2 inhibitors. Bioorg Med Chem Lett 19: 230-233.

*M:* Friberg A, Vigil D, Zhao B, Daniels RN, Burke JP, et al. (2013) Discovery of potent myeloid cell leukemia 1 (Mcl-1) inhibitors using fragment-based methods and structure-based design. J Med Chem 56: 15-30.

*N:* Tanaka Y, Aikawa K, Nishida G, Homma M, Sogabe S, et al. (2013) Discovery of potent Mcl‑1/Bcl-xL dual inhibitors by using a hybridization strategy based on structural analysis of target proteins. J Med Chem 56: 9635-9645.
