# Supplementary material for: Selectivity by Small-Molecule Inhibitors of Protein Interactions Can Be Driven by Protein Surface Fluctuations
Source: PLoS Comput Biol. 2015 Feb 23;11(2):e1004081. doi: 10.1371/journal.pcbi.1004081 (PMC4338137; doi:10.1371/journal.pcbi.1004081)
Supplement: S8 Table — This table shows the raw data from which the heatmap in Fig. 8 was created. (DOCX) [file pcbi.1004081.s017.docx]

| ***Active*** | |
| --- | --- |
| BRD4(1) | 0.83 |
| BRD3(2) | 0.84 |
| BRD3(1) | 0.95 |
| BRD4(2) | 0.70 |
| BRDT(1) | 0.65 |
|  | |
| ***Inactive*** | |
| TAF1L(2) | 0.57 |
| TIF1 | 0.64 |
| ATAD2 | 0.56 |
| SMARCA4 | 0.52 |
| BAZ2B | 0.55 |
| FALZ | 0.50 |
| PCAF | 0.55 |
| PB1(1) | 0.52 |
| TRIM28 | 0.48 |
| PB1(5) | 0.48 |

Table S8: Exemplar similarity of top (closest) pocket optimized structures to (+)-JQ1-BRD4(1) bound structure. This table shows the raw data from which the heatmap in Figure 8 was created.
