# Supplementary material for: Selectivity by Small-Molecule Inhibitors of Protein Interactions Can Be Driven by Protein Surface Fluctuations
Source: PLoS Comput Biol. 2015 Feb 23;11(2):e1004081. doi: 10.1371/journal.pcbi.1004081 (PMC4338137; doi:10.1371/journal.pcbi.1004081)
Supplement: S1 Text — This supporting text contains a complete description of methodology used, including PDB structures used in calculations. A description of pocket-opened conformations for each protein in our test set is also included in this text. (DOCX) [file pcbi.1004081.s018.docx]

## Supplementary Methods

### Rosetta software

Computational methods are implemented in the Rosetta software suite [18]. Calculations were carried out using git revision a27ab8d2408fb4a2 of the developer trunk source code.

A sample Rosetta command line used to identify target residues on chain A of an input.pdb using the Robetta ΔΔG calculations pasted into ddg.txt is as follows:

pocket_suggest_target_residues_by_ddg.linuxgccrelease ‑s input_pdb

–ddg_list ddg.txt –target_chain_list A

A sample Rosetta command line used to generate an exemplar is as follows:

make_exemplar.linuxgccrelease ‑s input_pdb ‑pocket_grid_size 12

‑pocket_filter_by_exemplar –pocket_static_grid ‑central_relax_pdb_num 108

### Dataset of Bcl‑2 family complexes

Structures that met any of the following criterion were excluded from the set of known Bcl‑2 family inhibitors: fragments below m.w. 250 Da, structures with multiple ligands in the binding site, structures that have the binding site defined by a (non-biologically relevant) homodimer, structures in which there was multiple occupancy of the inhibitor in the binding site, and fusion inhibitors that extended well out of the binding pocket. The complete list of complexes is presented as **Table S1**.

### PDB structures used in calculations

Unbound structures used as a starting point for “pocket opening” simulations were from the following PDB IDs: 1R2D (Bcl‑xL), 1G5M (Bcl‑2), 1WSX (Mcl‑1), 1MK3 (Bcl‑w), 1F16 (Bax), 2BID (Bid), and 1OHU (Ced9). Peptide-bound structures used as a starting point for “pocket opening” simulations were from the following PDB IDs: 1BXL (Bcl‑xL), 2XA0 (Bcl‑2), 2NL9 (Mcl‑1), and 3MK8 (Mcl‑1). In the case of NMR structures with multiple models, the first model was used.

Results for **Figure 8** were described for fifteen bromodomains comprising our test set: 2OSS (first bromodomain of BRD4), 2OO1 (second bromodomain of BRD3), 3HMH (first bromodomain of BRD3), 2OUO (second bromodomain of BRD4), 2RFJ (first bromodomain of BRDT), 3HMH (second bromodomain of TAF1L), 2YYN (TIF1), 3DAI (ATAD2), 2GRC (SMARCA4), 3G0L (BAZ2B, 2F6N (FALZ), 3GG3 (PCAF), 3IU5 (first bromodomain of PB1), 2RO1 (TRIM28), and 3G0J (fifth bromodomain of PB1). Comparisons were to 3MXF (the inhibitor-bound structure of the first bromodomain of BRD4). Data were collected for 5,000 independent simulations for each Bromodomain at the homologous target residues for the BRD4-JQ1 bound structure (residues 94 and 146 in 3MXF).

### Building ensembles of pocket-containing conformations

Ensembles of conformations were generated using the “relax” protocol in Rosetta with the pocket biasing potential as follows:

relax.linuxgccrelease ‑s input_pdb -relax:fast -pocket_max_spacing 12

‑pocket_zero_derivatives -pocket_psp false -pocket_sps -pocket_num_angles 2

-ex1 ‑ex1aro -ex2 -score:patch pocket.wts.patch -nstruct 1

-cst_fa_file constraints

The file pocket.wts.patch simply contains the text “pocket_constraint 1.0”, while the file constraints contains “Pocket 0.25 123:A,129:A”, where the target residues are defined as residues 123 and 129 in chain A of the PDB file.

Output structures were then subjected to a local energy minimization in the absence of the pocket biasing potential:

minimize.linuxgccrelease ‑s input_pdb

Conformations with very small pockets led to exemplars with a small number of atoms: exemplars with fewer than ten atoms were also removed from consideration. We also removed from consideration any conformations with energy more than 15 Rosetta Energy Units from those sampled in an analogous set of unbiased simulations.

### Generating “exemplars” to represent pockets

Exemplar generation occurs in the “pocket” protocol within Rosetta, and begins by defining the “deep pocket” volume as described previously [14].

The ideal locations of hydrogen bond acceptors (per the Rosetta energy function) are then identified. This is accomplished by identifying all solvent exposed hydrogen bond donors, then calculating the location of an ideal hydrogen bond acceptor for each: an ideal acceptor lies 2.75 Å from the donor atom, on the vector that passes through the donor “base” atom and the hydrogen atom. Acceptor locations that touch the target pocket and do not clash with the protein are included in the exemplar.

Ideal hydrogen bond donors are then identified by iterating over all solvent exposed hydrogen bond acceptors: an ideal donor lies 2.75 Å from the acceptor atom, though its location also depends on the bond angles, Φ and Θ, of the local coordinate system defined by the acceptor atom (origin), its base atom (setting the direction of the X axis) and the hybridization of the accepting group [S1]. For each accepting group, potential donor locations are tested in search of one that touches the target pocket and does not clash with the protein; if found, this donor location is added to the exemplar.

Next, the deep pocket points are scanned to identify points whose 26 neighboring pocket grid points and three next-nearest neighbors in the positive X, Y, and Z directions are all deep pocket points not yet included in the exemplar. When such a point is found, that point is included in the exemplar as a carbon atom, and 26 neighboring grid points are excluded from further searching.

Exemplar atoms within 3.5 Å of one another are then clustered together; any clusters that are not in direct contact with the “target” residue used to define the deep pocket are removed.

Finally, the exemplar is printed to a file using PDB format.

### Treatment of donors/acceptors in ROCS

The ROCS software is built to use “real” compounds as input, rather than exemplars. Accordingly, ROCS assigns hydrogen bond donors/acceptors upon reading in the template molecule. Oxygen and nitrogen atoms are identified by ROCS as either acceptors or donors (or both) based on their connectivity.

Since our exemplars lack this connectivity, it was necessary to define new atom types that are forced to serve as a pre-determined donor/acceptor regardless of context. To do this, we edited the beryllium and neon element types in ROCS such that these served as obligate hydrogen bond donors and acceptors, respectively. We then doubled the scale of the radius to allow for near optimal hydrogen bonding. Beryllium and neon were chosen because of their comparable size to nitrogen and oxygen, as well as their paucity in drug-like compounds.

When comparing exemplars, we used the modified chemical force field and included chemical complementarity only in the final scoring phase (i.e. only shape considerations were included in the optimization phase).

### Comparison of chemical structures

Chemical structures were performed by OpenBabel [S2,S3] using FP2 fingerprints. FP2 fingerprints index the small molecule by identifying linear segments of one to seven atoms that are then indexed in a 1024 bit vector [S2,S3]. Comparisons are scored using the Tanimoto coefficient (Jaccard index): the ratio of number of chemical moieties common to both molecules to the total number of chemical moieties used.

### Numerical analysis

Spearman rank correlation coefficients and their significance (**Figure 2**) were also computed in the R statistical computing environment [S4]. Multidimensional scaling analysis was also carried out in R [S4], using the cmdscale function. The reported statistical significance for the ROC plot (**Figure 7b**) was calculated using a one‑tailed P-value with null hypothesis that the area under the curve is 0.5 [S5].

## Supplementary References

S1. Kortemme T, Morozov AV, Baker D (2003) An orientation-dependent hydrogen bonding potential improves prediction of specificity and structure for proteins and protein-protein complexes. J Mol Biol 326: 1239-1259.

S2. The Open Babel Package, version 2.3.1. http://www.openbabel.org (referenced Oct 2011).

S3. O'Boyle NM, Banck M, James CA, Morley C, Vandermeersch T, et al. (2011) Open Babel: An open chemical toolbox. Journal of Cheminformatics 3: 33.

S4. R Core Team (2014) R: A Language and Environment for Statistical Computing. Vienna, Austria: R Foundation for Statistical Computing.

S5. Hanley JA, McNeil BJ (1982) The meaning and use of the area under a receiver operating characteristic (ROC) curve. Radiology 143: 29-36.
